# Silkworms suppress the release of green leaf volatiles by mulberry leaves with an enzyme from their spinnerets

**DOI:** 10.1038/s41598-018-30328-6

**Published:** 2018-08-09

**Authors:** Hiroki Takai, Rika Ozawa, Junji Takabayashi, Saki Fujii, Kiriko Arai, Ryoko T. Ichiki, Takao Koeduka, Hideo Dohra, Toshiyuki Ohnishi, Sakura Taketazu, Jun Kobayashi, Yooichi Kainoh, Satoshi Nakamura, Takeshi Fujii, Yukio Ishikawa, Takashi Kiuchi, Susumu Katsuma, Masayoshi Uefune, Toru Shimada, Kenji Matsui

**Affiliations:** 1 Division of Agricultural Sciences, Graduate School of Sciences and Technology for Innovation, Yamaguchi University, Yamaguchi, 753-8515 Japan; 2 Laboratory of Insect Genetics and Bioscience, Graduate School of Agricultural and Life Sciences, The University of Tokyo, Tokyo, 113-8657 Japan; 3 Center for Ecological Research, Kyoto University, Otsu Shiga, 520-2113 Japan; 4 Department of Biological Chemistry, Faculty of Agriculture, Yamaguchi University, Yamaguchi, 753-8515 Japan; 5 Japan International Research Centre for Agricultural Sciences, Tsukuba, Ibaraki, 305-0851 Japan; 6 Research Institute of Green Science and Technology, Shizuoka University, Shizuoka, 422–8529 Japan; 7 College of Agriculture, Academic Institute, Shizuoka University, Shizuoka, 422–8529 Japan; 8 Department of Biological and Environmental Sciences, Faculty of Agriculture, Yamaguchi University, Yamaguchi, 753-8515 Japan; 9 Faculty of Life and Environmental Sciences, University of Tsukuba, Tsukuba Ibaraki, 305-8572 Japan; 10 Laboratory of Applied Entomology, Graduate School of Agricultural and Life Sciences, The University of Tokyo, Tokyo, 113-8657 Japan; 11 Department of Agrobiological Resources, Faculty of Agriculture, Meijo University, Nagoya, Aichi 468-8502 Japan

## Abstract

In response to herbivory, plants emit a blend of volatile organic compounds that includes green leaf volatiles (GLVs) and terpenoids. These volatiles are known to attract natural enemies of herbivores and are therefore considered to function as an indirect defense. Selection should favor herbivores that are able to suppress these volatile emissions, and thereby make themselves less conspicuous to natural enemies. We tested this possibility for silkworms, which were observed to leave secretions from their spinnerets while feeding on mulberry leaves. When we ablated the spinnerets of silkworms, no secretions were observed. Leaves infested by intact silkworms released smaller amounts of GLVs than leaves infested by ablated silkworms, indicating that the spinneret secretion suppressed GLV production. This difference in GLV emissions was also reflected in the behavioral response of *Zenillia dolosa* (Tachinidae), a parasitoid fly of silkworms. The flies laid fewer eggs when exposed to the volatiles from intact silkworm-infested leaves than when exposed to the volatiles from ablated silkworm-infested leaves. We identified a novel enzyme in the secretion from the spinneret that is responsible for the GLV suppression. The enzyme converted 13(*S*)-hydroperoxy-(9*Z*,11*E*,15*Z*)-octadecatrienoic acid, an intermediate in the biosynthetic pathway of GLVs, into its keto-derivative in a stereospecific manner. Taken together, this study shows that silkworms are able to feed on mulberry in a stealthy manner by suppressing GLV production with an enzyme in secretions of their spinnerets, which might be a countermeasure against induced indirect defense by mulberry plants.

## Introduction

Accumulating evidence shows that some plants respond to the cues in herbivores’ secretions by producing herbivore-induced plant volatiles (HIPVs) that attract carnivorous natural enemies of currently infesting herbivores^1–4^. When the attraction of carnivores by HIPVs benefits the plants, the production of HIPVs is considered to be induced indirect defense of plants against herbivores^1–4^. Among HIPVs, the production of volatile terpenoids is induced by plants in response to the combination of the mechanical damage of plant tissues that is the inevitable consequence of herbivory and by secretions from herbivores^1^. In contrast, the production of green leaf volatiles (GLVs), another class of HIPVs, is induced primarily by mechanical damage caused by herbivory^5,6^. However, here again, there is a case where the production of GLVs is modified by secretions of infesting herbivores: an oral secretion from *Manduca sexta* (Sphingidae) larvae decreased the (*Z*)/(*E*) ratio of GLVs emitted from *Datura wrightii* and *Nicotiana attenuata*^7,8^. The larvae-induced decrease in the (*Z*)/(*E*) ratio of GLVs reduced oviposition by female *M. sexta* moth^7^, but the decrease unexpectedly resulted in higher foraging efficiency of predators (*Geocoris* spp., Geocoridae) on the larvae^8^.

Secretions deposited by herbivorous arthropods on plant tissues during feeding, in some cases, suppress plant defenses against herbivores^9^. For example, *Theroa zethus* (Notodontidae) larvae produce acid secretions from the ventral eversible gland that created furrows, thereby physically suppressing the release of poisonous latex from the veins of Euphorbiaceae^10^. Furthermore, certain chemicals in herbivore secretions have been reported to suppress defense responses in plants (effectors)^9^. The larvae of *Helicoverpa zea* (Noctuidae) and *Spodoptera exigua* (Noctuidae), for example, secrete saliva containing an enzyme, glucose oxidase, from their spinnerets that suppresses induced direct defenses in plants^11,12^. Glucose oxidase in *S. exigua* larvae attenuates jasmonic acid (JA) and ethylene formation in plants after herbivore damage, thus suppressing the defense response controlled by JA and ethylene signaling pathways^12^. Effector-like proteins found through secretome analysis in the salivary glands of *Tetranychus urticae* (Tetranychidae) and *T. evansi* (Tetranychidae) suppressed plant defenses downstream of salicylic acid (SA), and *T. urticae* performance improved on *Nicotiana benthamiana* leaves transiently expressing some of the proteins^13^.

Since induction of the carnivore-attractive HIPV production by herbivores is maladaptive to the herbivores, there should be selection for herbivores to suppress such induction to make themselves more inconspicuous to natural enemies. Such suppression has been reported in a few plant-herbivore systems. In tobacco (*Nicotiana tabacum*), feeding by *Heliothis virescens* (Noctuidae) caterpillars with intact spinnerets induced lower amounts of volatile terpenoids compared to feeding by caterpillars with ablated spinnerets^14^. Oral secretion of *M. sexta* promoted formation of a subset of volatiles and suppressed formation of another subset of volatiles in *N. attenuata*^15^. Regurgitant of *Pieris rapae* (Pieridae) larvae suppressed formation of a subset of GLVs in *Arabidopsis thaliana*^16^. Based on these results, it can be assumed that some herbivores have acquired a countermeasure against HIPV-mediated induced indirect defense of plants; however, the factor(s) involved in the suppression of HIPVs and its mechanism of action have not been clarified and the ecological functions of these changes in volatiles in insect-plant interactions have not been tested. Here, we examined the effects of spinneret secretions on plant defenses using a tritrophic system of mulberry plants (*Morus alba*), silkworms (*Bombyx mori*, Bombycidae), and one of their natural enemies, a parasitoid fly *Zenillia dolosa*. Silkworms deposit silken thread through spinnerets on the surface of leaves while feeding, and the secretions from spinnerets deposited on the fed-edge of mulberry leaves suppressed production of GLVs among other groups of volatiles in mulberry leaves. The parasitoid fly *Z. dolosa* laid fewer eggs in the presence of suppressed amounts of volatiles. Further, we examined the factor responsible to the suppression in the secretion.

## Results

### Secretions from silkworm spinneret

**Figure 1:**
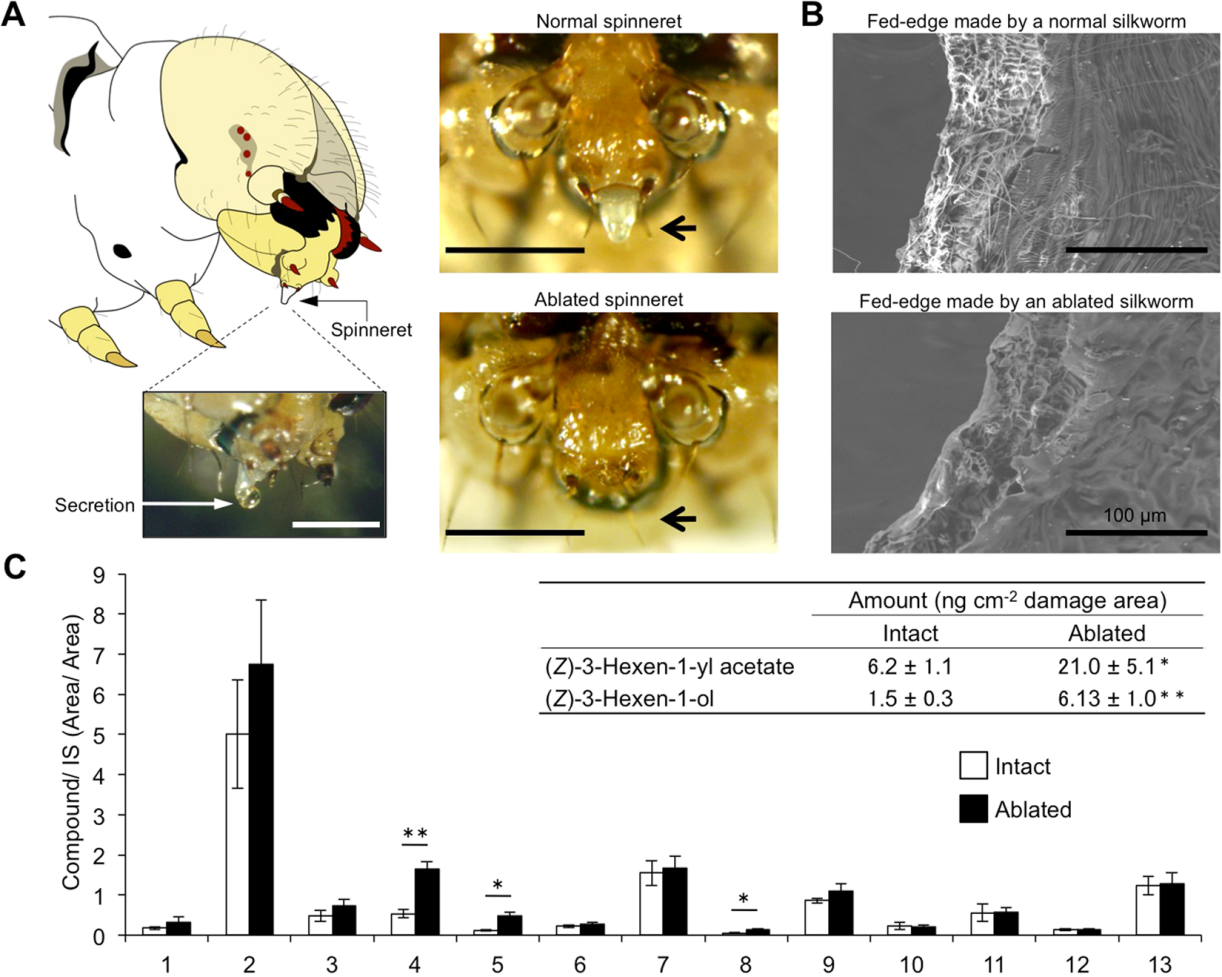
Secretions from the spinneret deposited on the leaf edge during feeding suppressed formation of green leaf volatiles. (**A**) A drawing of the head part of *Bombyx mori* larva (left top). A droplet was secreted from the spinneret after touching it with forceps (left bottom). Photos of a spinneret from *Bombyx mori* (right top) and the spinneret after ablation (right bottom) are shown. Bars are 50 µm. (**B**) Scanning electron micrographs of sections of mulberry leaves that were damaged by a silkworm with intact spinnerets (top) and a silkworm without spinnerets (bottom). (**C**) Amounts of volatile compounds emitted from mulberry leaves infested with silkworms with intact spinnerets (white bars) or without spinnerets (black bars). Compounds 1: (*Z*)-β-ocimene, 2: (*E*)-β-ocimene, 3: geranyl nitrile, 4: (*Z*)-3-hexen-1-yl acetate, 5: (*Z*)-3-hexen-1-ol, 6: unknown monoterpene 1, 7: unknown monoterpene 2, 8: (*Z*)-3-hexen-1-yl butanoate, 9: linalool, 10: α-farnesene, 11: methyl salicylate, 12: 2-methyl-3-buten-2-ol, and 13: (*Z*)-*p*-menth-2,8-dien-1-ol. Those identified by authentic compounds are underlined. Inset: amounts of (*Z*)-3-hexen-1-yl acetate and (*Z*)-3-hexen-1-ol quantified from corresponding calibration curves. Means with error bars are shown (SE, *n* = 5). Significant differences between the amounts of volatiles emitted after infestation with the intact and the ablated spinnerets are indicated by asterisks (*P < 0.05; **P < 0.01; Welch’s *t*-test).

While feeding on mulberry leaves, the silkworm secreted droplets through its spinneret. The silkworm spun the thread from the droplets while continuously moving its head backward and forward and feeding (Supplemental Fig. 1, Supplemental video). A droplet was also secreted from the spinneret when it was physically stimulated with forceps (Fig. 1A). Scanning electron microscopy (SEM) revealed that threads produced by the silkworm were deposited on the wounded edge surface (Fig. 1B).

To further clarify the origin of the threads on the fed edge, the spinnerets of the fifth instars were carefully cauterized (Fig. 1A). We then offered mulberry leaves to the spinneret-ablated silkworms and control (intact) silkworms. The leaf areas consumed within 6 h by the spinneret-ablated and intact silkworms were not significantly different [*t*-test (*n* = 5), *t* = 0.3397, df = 8, *P* = 0.7429] (Supplemental Fig. 2), indicating that the ablation had minimal effect on consumption rate, at least for 6 h. An SEM photograph showed no threads on the surface of the fed edge made by the spinneret-ablated silkworms (Fig. 1B).

### Effects of ablation of spinnerets on volatiles from plants infested by silkworms

We analyzed the volatiles from mulberry leaves that were either uninfested or infested with silkworms with intact or ablated spinnerets. Volatile compounds were hardly detected from uninfested mulberry leaves (Supplemental Fig. 3). The mulberry leaves that were infested by silkworms of either intact or spinneret-ablated emitted 13 volatile compounds (Fig. 1C). Ten of the volatile compounds (including nine volatile terpenoids) did not differ significantly in amounts between the two odor sources [Welch’s *t*-test (*n* = 5); (*Z*)-β-ocimene (*t* = 0.8245, df = 7.7489, *P* = 0.4343), (*E*)-β-ocimene (*t* = 1.1607, df = 4.6091, *P* = 0.3023), geranyl nitrile (*t* = 1.2220, df = 7.9611, *P* = 0.2566), linalool (*t* = 1.2492, df = 5.0184, *P* = 0.2667), α-farnesene (*t* = 0.2681, df = 5.3565, *P* = 0.7987), methyl salicylate (*t* = 0.034, df = 6.6742, *P* = 0.9739), 2-methyl-3-buten-2-ol (*t* = 0.0109, df = 7.807, *P* = 0. 9916), (*Z*)-*p*-menth-2,8-dien-1-ol (*t* = 0.1749, df = 7.8485, *P* = 0.8656), unknown monoterpene 1 (*t* = 0.8033, df = 6.9795, *P* = 0.4483), and unknown monoterpene 2 (*t* = 0.2567, df = 7.9995, *P* = 0.8039)]. By contrast, the leaves that were infested by the spinneret-ablated silkworms emitted significantly higher amounts of the three GLVs when compared with those from leaves infested by normal silkworms [Welch’s *t*-test (*n* = 5); (*Z*)-3-hexen-1-yl acetate (*t* = 4.9562; df = 5.8523; *P* = 0.0027), (*Z*)-3-hexen-1-ol (*t* = 4.1434; df = 4.4123; *P* = 0.0117), and (*Z*)-3-hexen-1-yl butanoate (*t* = 2.737; df = 4.2616; *P* = 0.0486)] (Fig. 1C). These data suggest that the secretions produced by the silk glands and delivered via the spinnerets to the wounded edge negatively affected the production of the three GLVs.

### Response of parasitoid flies to volatiles

**Figure 2:**
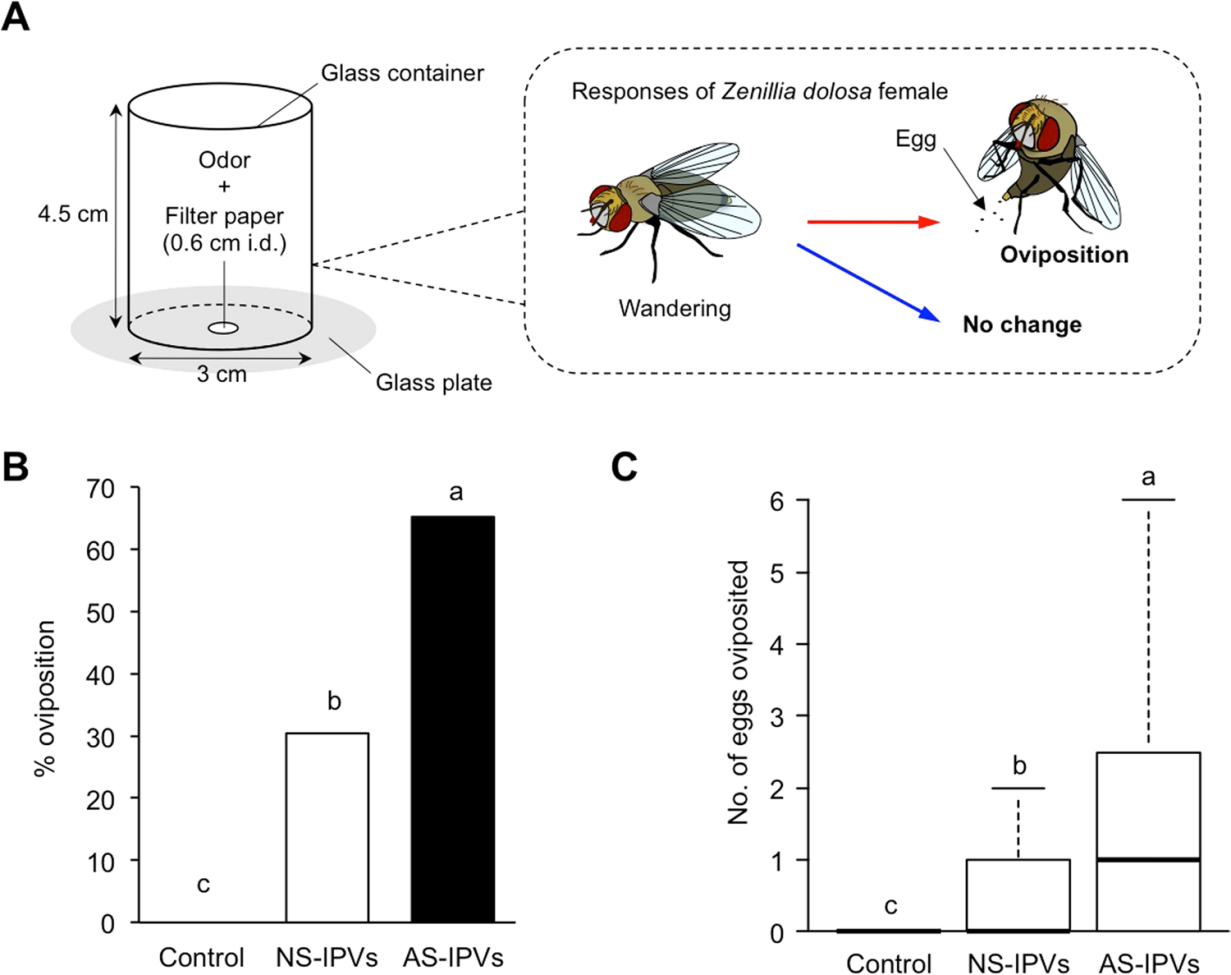
The oviposition response of *Zenillia dolosa* females to the exposure of volatiles emitted from mulberry leaves infested with silkworms with or without spinnerets. (**A**) Experimental set-up used in this study. (**B**) Oviposition rate of *Z. dolosa* females (Fisher’s exact test followed by Holm’s *P*-value adjustment, *P* < 0.05). Dichloromethane was used as the control. NS-IPVs; normal silkworm-induced plant volatiles, AS-IPVs; ablated silkworm-induced plant volatiles. Different letters above the bars indicate significant differences among treatments (*n* = 23). (**C**) Number of eggs (±SE) deposited by *Z. dolosa* (*P* < 0.05, Steel-Dwass test, *n* = 23). The length of each box plot shows the interquartile range; the centerline marks the median, and box edges are at the first and third quartiles. Whiskers show the highest and lowest values within a distance of 1.5 times the interquartile range from box edges.

*Zenillia dolosa* is a micro-type parasitoid fly species that parasitizes the larvae of several lepidopteran species, including silkworms^17^. Female *Z. dolosa* have been shown to be more attracted to an artificially damaged plant than to an intact plant to lay eggs on the leaf surfaces near the damaged site, possibly responding to the volatiles emitted as a result of the mechanical damage to the plant^18^. To evaluate the effect of amounts of volatiles on oviposition behavior of *Z. dolosa* females, we collected volatiles from mulberry leaves infested by either intact or spinneret-ablated silkworms and used them as odor sources (Fig. 2A). *Z. dolosa* females showed no oviposition behavior when exposed to the control (a piece of filter paper with a pure solvent) (Fig. 2B,C). The proportion of *Z. dolosa* females that showed oviposition responses in the presence of volatiles from leaves infested by normal silkworms (30.4%) was significantly lower than with leaves infested by the spinneret-ablated silkworms (65.2%) (Fisher’s exact test followed by Holm’s *P*-value adjustment, *P* = 0.0377) (Fig. 2B). The number of eggs oviposited in the presence of volatiles from leaves infested by normal silkworms (median = 0, first quartile = 0, third quartile = 1) was also significantly lower than that in the presence of volatiles from leaves infested by the spinneret-ablated silkworms (median = 1, first quartile = 0, third quartile = 3) (Steel-Dwass test, *P* = 0.0279) (Fig. 2B).

### Inhibition by silk gland extract of formation of GLVs

**Figure 3:**
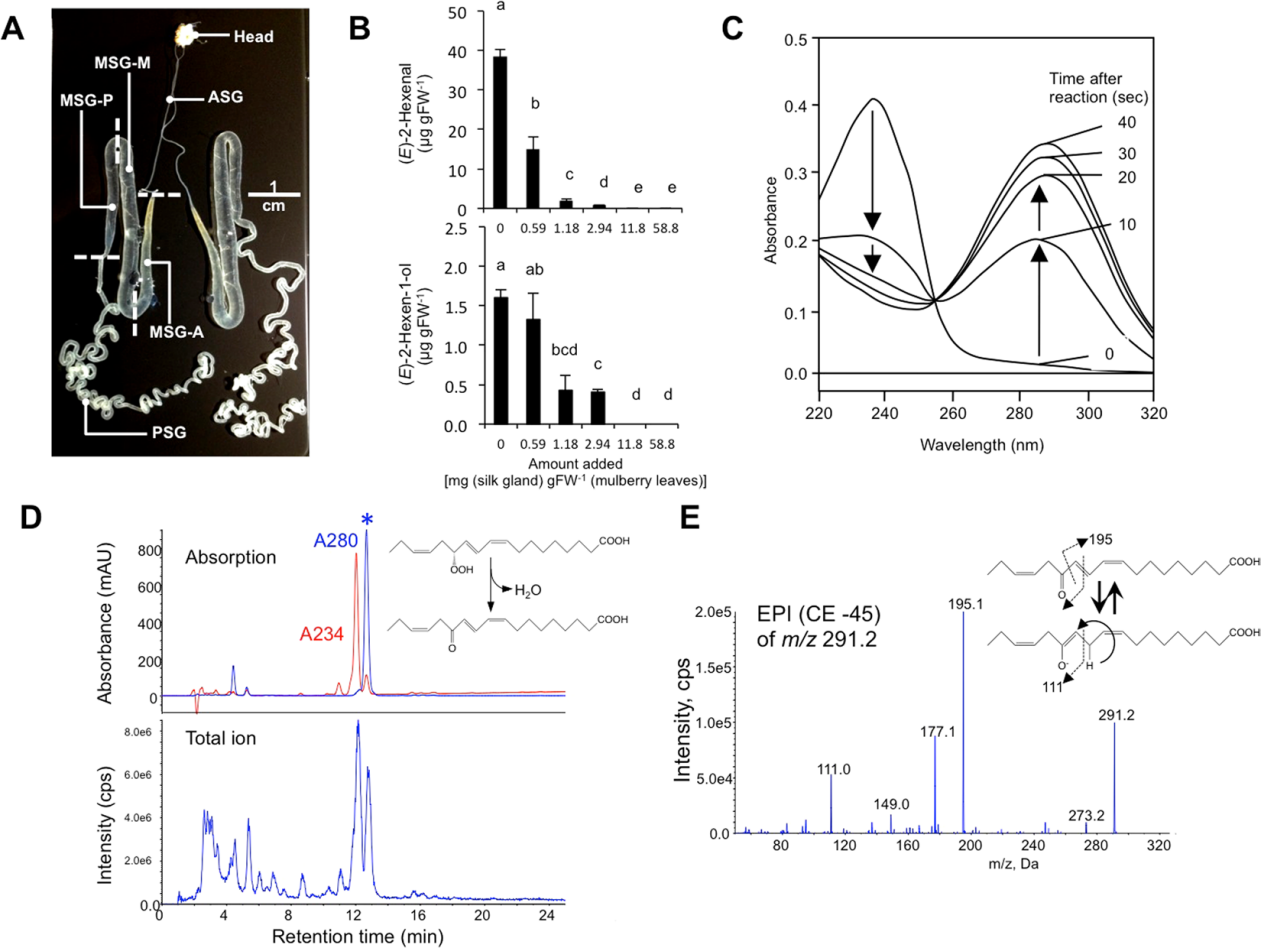
The silk gland extract suppressed formation of green leaf volatiles through conversion of fatty acid hydroperoxide into its keto derivative. The mulberry leaf powder was homogenized in the presence of silk gland extract equivalent to 0–58.8 mg wet weight of each part of the silk gland (per 1 g fresh weight of leaves) and incubated for 10 min to facilitate enzyme reaction to form volatiles. (**A**) Silk gland used for extraction. (**B**) The amounts of (*E*)-2-hexenal and (*E*)-2-hexen-1-ol formed in the absence or presence of the silk gland extract that was obtained from the anterior part of the silk gland (consisting of the anterior silk gland and the anterior part of middle silk gland, ASG + MSG-A). Averages with error bars (SE, *n* = 3, technical replicate) are shown. The lowest amount of the silk gland extract (0.59 mg) corresponded to 0.016 [ASG + MSG-A] equivalent of that derived from one silkworm. Different letters above the bars indicate significant differences among treatments for each extract (*P* < 0.05, GLM following Holm’s *P*-value adjustment). (**C**) Overlay of UV spectra of the reaction mixture consisting of the silk glands with 20 µM of 13*S*-HPOT. The reaction was scanned from 220 to 320 nm at 0, 10, 20, 30, and 40 sec after substrate addition. (**D**) HPLC-MS/MS analyses of the products formed by the silk gland extract from 13*S*-HPOT. Chromatograms with absorption at 280 nm (blue) and 234 nm (red) (upper chromatogram) and total ion chromatogram obtained with enhanced MS mode (lower chromatogram) are shown. The peak with an asterisk had a spectrum with λmax at 280 nm. The precedent peak is 13*S*-HPOT. (**E**) MS profile obtained with the peak with asterisk in (**D**) with enhanced product ion mode with the collision energy of −45 V with the molecular ion of *m/z* 291.2. The possible fragmentation patterns are also shown. Proposed reaction of the enzyme to form 13-oxo-(9*Z*,11*E*,15*Z*)-octadecatrienoic acid (13-OTE) from 13*S*-HPOT is shown in inset of d.

GLVs are rapidly produced via the phytooxylipin pathway when leaf tissues are disrupted^5,6,19^. To determine whether the silk gland components affected the biosynthesis of GLVs, we prepared buffer extracts from the anterior part of the silk gland (consisting of the anterior silk gland and anterior part of the middle silk gland, ASG + MSG-A) (Fig. 3A), and the mulberry leaves were completely disrupted with freeze-thaw treatments to induce GLV formation in the presence of the extract. When we analyzed the methyl *tert*-butyl ether extract of the mulberry leaves after freeze-thaw disruption in the absence of silk gland extract, a subset of GLVs, i.e. (*E*)-2-hexenal and (*E*)-2-hexen-1-ol, was detected. However, the presence of the silk gland extracts prepared from ASG + MSG-A extensively suppressed their formation in a dose-dependent manner (Fig. 3B). Such a suppression was not observed when the extracts from the middle to posterior part of middle silk gland MSG(M-P) and those from the posterior silk gland (PSG) were used (Supplemental Fig. 3).

### Identification of factor involved in suppression of GLVs

Within seconds after the damage to plant tissues, linolenic acid is oxygenated by lipoxygenase (LOX) to form 13-hydroperoxy-(9*Z*,11*E*,15*Z*)-octadecatrienoic acid (13*S*-HPOT), which is subsequently cleaved by hydroperoxide lyase (HPL) to form (*Z*)-3-hexenal (Supplemental Fig. 4)^5,6^. When 13*S*-HPOT reacted with the silk gland extract, the characteristic absorbance of the 13*S*-HPOT (with λmax at 234 nm) decreased with an accompanying rise in the absorbance at 280 nm (Fig. 3C). Analysis with high-performance liquid chromatography coupled with tandem mass spectrometry (HPLC-MS/MS) of the products enabled us to speculate on the chemical structure of the product based on the *m/z* values of 291.2 ([M − H]^−^), 195.1 (carboxyl terminal fragment after cleavage at C12-C13 bonding), and 111.0 (*ω*-terminal fragment after cleavage at C11-C13 bonding after keto-enol tautomerism)^20^ to be 13-oxo octadecatrienoic acid (13-OTE) (Fig. 3D,E). The chemical structure of 13-OTE was confirmed with the authentic 13-OTE. The results indicated that the silk gland extract converted 13-HPOT into 13-OTE (Fig. 3D, *inset*).

We purified the factor involved in the suppression of GLVs by focusing on the conversion of 13*S*-HPOT into 13-OTE (Supplemental Table 1). During the two chromatography steps, the activity eluted as a single peak with a substantial yield. SDS-PAGE revealed that the purified factor was a single protein of 32 kDa (Supplemental Fig. 5A). Thus, the purified protein was an enzyme that converted 13*S*-HPOT to 13-OTE with maximal activity at pH 9.0–9.5 (Supplemental Fig. 5B). The enzyme needed no cofactor. The enzyme obeyed Michaelis–Menten kinetics with 13*S*-HPOT (Supplemental Fig. 5C), and the *K*_m_, *V*_max_, and *k*_cat_ values were estimated to be 1.13 µM, 10.7 nmol sec^−1^ mg^−1^, and 3.5 × 10^5^ sec^−1^, respectively. After mixing with 13*S*-HPOT, the purified enzymes immediately started consuming the 13*S*-HPOT, but the reaction rate gradually decreased and almost ceased before all of the available substrate was consumed (Supplemental Fig. 5D). The addition of the fresh enzyme into the mixture restarted the reaction at a rate similar to that of the initial reaction but the secondary addition of 13*S*-HPOT did not restart the reaction (Supplemental Fig. 5E,F). The inactivation of the enzyme proceeded in a time-dependent manner (Supplemental Fig. 5D, *inset*). Under the standard reaction conditions [110 ng (3.4 pmol) of purified enzyme and 20 nmol of 13*S*-HPOT], the enzyme catalyzed the reaction 1.8 × 10^4^ times before inactivation.

The substrate specificity of purified enzyme was examined by incubating a mixture of HPOs of linoleic acid (hydroperoxy-octadecadienoic acids: HPODs) prepared by auto-oxidation. The HPOD mixture comprised eight isomers, including two positional isomers (9- and 13-HPOs), two geometrical isomers (*Z* and *E* forms with the double bond distal to the hydroperoxy group) and two enantiomers (*R* and *S*). Incubation of the HPOD mixture with the purified enzymes resulted in a decrease in the amount of 13-hydroperoxy-(9*Z*,11*E*)-octadecadienoic acid and a concomitant increase of 13-oxo octadecadienoic acid (13-ODE) (Supplemental Fig. 6A). By contrast, the amounts of the other isomers hardly changed. When the 13-hydroperoxy-(9*Z*,11*E*)-octadecadienoic acid remaining after the enzymatic reaction was delivered to the chiral phase HPLC, a significant decrease in the peak corresponding to the 13(*S*)-isomer was detected (Supplemental Fig. 6B). In summary, the enzyme had a stereospecificity to 13(*S*)-hydroperoxy-(9*Z*,11*E*)-octadecadienoic acid. Hereafter, we refer to the enzyme as *Bombyx mori* fatty acid hydroperoxide dehydratase (BmFHD).

### Molecular cloning of gene

The internal amino acid sequences obtained from the purified protein showed a complete identity with uncharacterized protein LOC101739721 (gI|512894896) in the NCBInr database (Supplemental Fig. 7). Searching the genome and cDNA sequences in SilkBase (http://silkbase.ab.a.u-tokyo.ac.jp) indicated that a gene (BGIBMGA006282-TA) located on the sixth chromosome encoded a protein of 289 amino acids (DDBJ accession no; LC259005). Based on InterProScan 5, a signal peptide consisting of 19 amino acids was predicted at the *N*-terminal end. The amino acid sequence of BmFHD showed no homology to any oxygenases or flavin enzymes reported to date. A Conserved Domain Search in the NCBI database, PROSITE, and Pfam indicated no putative conserved domains in BmFHD.

We expressed a recombinant protein encoded by *BmFHD* in a *B. mori* cell line, BmN4. The supernatants of BmN4 cell cultures, infected with recombinant baculoviruses harboring the cDNA sequence of *BmFHD*, consumed 13*S*-HPOT and converted it into 13-OTE (Supplemental Fig. 8). No activity was detected in the cell homogenate. These results indicate that the cDNA encodes the BmFHD enzyme. The signal sequence at the *N*-terminal end effectively secreted the protein from the BmN4 cells.

### Expression of *BmFHD* in different tissues and developmental stages

**Figure 4:**
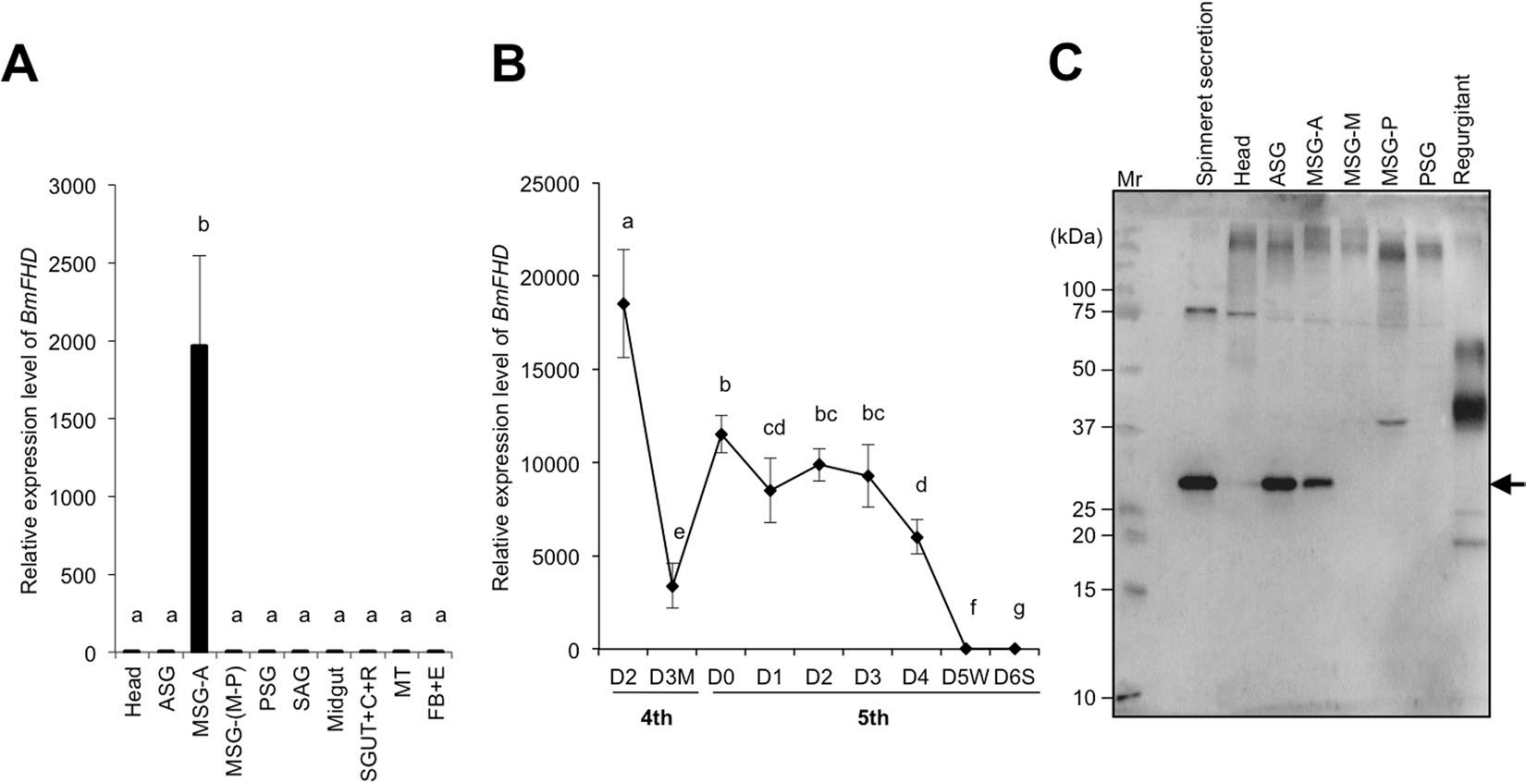
Temporal and spatial expression profiles of *BmFHD*. (**A**) Tissue-specific expression profile of *BmFHD* in the fifth instars (third day, N4 strain). The instar was dissected into the head, anterior silk glands (ASG), anterior part of middle silk glands (MSG-A), middle-to-posterior parts of middle silk glands [MSG(M-P)], posterior silk glands (PSG), salivary glands (SAG), midgut (MGUT), small gut plus colon plus rectum (SGUT + C + R), Malpighian tube (MT) and fat body plus epidermis (FB + E). The means are shown with SE (*n* = 3). Different letters above the bars indicate significant differences among samples (*P* < 0.05, GLMM followed by Holm’s *P*-value adjustment). (**B**) The stage-specific expression profile of *BmFHD* in the middle silk glands (MSG-A), from the third day of the fourth instar to the sixth day of fifth instar (spinning stage). M, molting stage; W, wandering stage; S, spinning stage. For the RT-PCR analysis, *Bombyx mori ribosomal protein 49* (*Bmrp 49*) was used as an internal control. The means are shown with SE (*n* = 4). Different letters above the bars indicate significant differences among samples (*P* < 0.05, GLM followed by Holm’s *P*-value adjustment). (**C**) Immunoblot analysis to monitor the occurrence of the BmFHD protein in various organs and secretions at the third day of fifth instars. Lanes 1: a protein marker (Mr); 2: secretions from spinnerets; 3: head; 4: ASG; 5: MSG-A; 6: MSG-M; 7: MSG-P; 8: PSG; 9: oral regurgitant. BmFHD is indicated with the arrow. For each lane, 1 µg of protein was applied. An uncropped full-length image is shown.

The *BmFHD* gene was expressed predominantly in the anterior part of the middle silk glands (MSG-A) among the ten different parts dissected from the 3-day, fifth instar silkworms (Fig. 4A). The temporal expression profile of the *BmFHD* gene in MSG-A indicated that the transcript of the *BmFHD* gene was detected in the late stage of the fourth instar, but decreased during the fourth molting stage (on the third day of the fourth instar) (Fig. 4B). The transcript of *BmFHD* reappeared at the beginning of the fifth instar and remained at a high level until the third day of the fifth instar. Thereafter, the amount of transcript decreased until it was undetectable by the fifth (the wandering stage) and sixth (the spinning stage) days of the fifth instar.

When the protein extract from each part of the silk gland part was subjected to immunoblot analysis using antibodies raised against an internal peptide sequence of BmFHD, an intense signal was observed for MSG-A where the gene expression was evident (Fig. 4C). At the same time, a substantial signal was detected for the protein extract from the anterior silk glands (ASG) and, more importantly, from the secretion collected from the spinneret tip. The amount of BmFHD protein deposited by the fifth instars of silkworms on the fed edge of mulberry leaves was estimated with immunoblot analysis on the rinse collected from the fed edge (Supplemental Fig. 9A,B). The amount of BmFHD on the fed edge was calculated to be 32.1 ± 4.8 µg per g fresh weight (average ± standard error; *n* = 6). BmFHD was not detected on the fed edge when ablated silkworms fed on the leaves. When mulberry leaves were ruptured in the presence of purified BmFHD protein, significant suppression of (*E*)-2-hexenal formation was evident with 3.4 µg of purified BmFHD protein per g fresh weight of leaf (Supplemental Fig. 9C).

### Phylogenetic analysis of BmFHD

**Figure 5:**
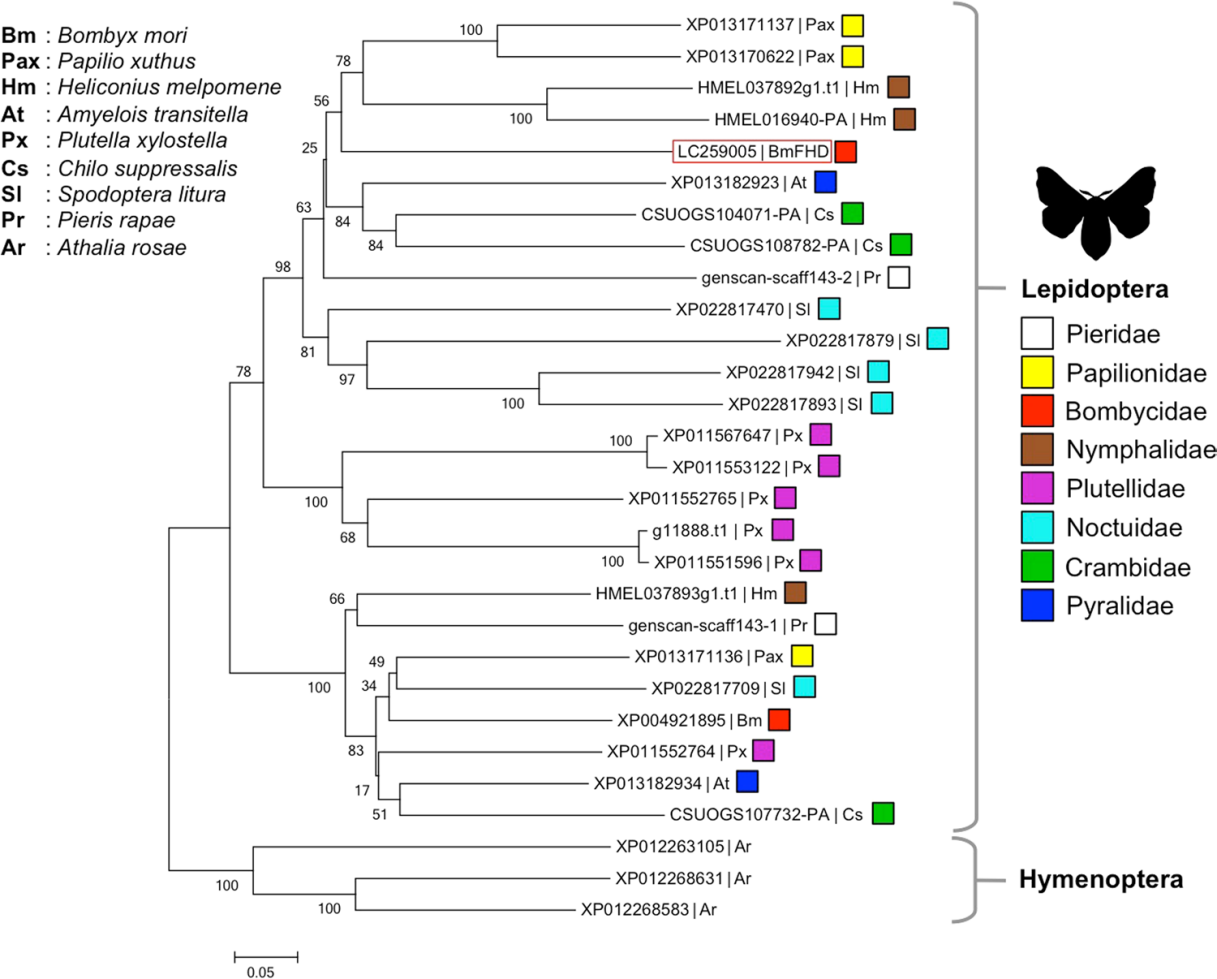
Phylogenetic tree made with BmFHD and its homologs. The TBLASTN analysis with BmFHD protein sequence (DDBJ accession number; LC259005) as a query was carried out with genome sequences of *Bombyx mori*, *Papilio xuthus*, *Heliconius melpomene*, *Amyelois transitella*, *Plutella xylostella*, *Chilo suppressalis*, *Spotoptera litura*, and *Pieris rapae*, and the sequences with homology to BmFHD were chosen to construct the tree. The neighbor-joining tree was generated with MEGA5. The scale bar indicates the evolutionary distance between groups. Values given at each node correspond to bootstrap values (1,000 replicates). The sequences from *Athalia rosae* showing slight homology to BmFHD (XP_012263105, XP_012268631, and XP_012268583) were used as outgroups.

TBLASTN analysis with genome sequences of lepidopteran insects, *Papilio xuthus* (Papilionidae), *Heliconius melpomene* (Nymphalidae), *Amyelois transitella* (Pyralidae), *Plutella xylostella* (Plutelidae), *Chilo suppressalis* (Crambidae), *Spodoptera litura* (Noctuidae), and *Pieris rapae* (Pieridae), showed that FHD-like genes occur in other lepidopteran insects (30–48% identity with an E value of 1.7 × 10^−32^ to 7.7 × 10^−98^ at the protein level) (Fig. 5, Supplemental Fig. 10). Genes showing slight homology to BmFHD (27–30% identity with an E value of 5.0 × 10^−16^ to 3.0 × 10^−22^ at the protein level) were also found in a hymenopteran, *Athalia rosae* (Tenthredinidae) (Fig. 5). No homologous sequence was found in the genome sequences of insect species such as the fruit fly (*Drosophila melanogaster*: Diptera), the African malaria mosquito (*Anopheles gambiae*: Diptera) and the pea aphid (*Acyrthosiphon pisum*: Hemiptera).

## Discussion

Almost all terrestrial plants have the ability to form and emit GLVs in response to mechanical damage caused by herbivores^5,6^. The formation and emission commence within seconds after tissue damage and are generally terminated within several minutes^19^. Thus, GLVs themselves bear information about the presence of plants under attack. The emission of GLVs as well as other groups of volatile compounds from plants might be one of the cues used by parasitoids and predators to find their hosts/prey that are actively feeding on plants. Several parasitoids and predators use GLVs in their foraging^1,3,4,21^. In the present study, mulberry leaves produced higher amounts of GLVs [(*Z*)-3-hexen-1-ol, (*Z*)-3-hexen-1-yl acetate and (*Z*)-3-hexen-1-yl butanoate] when they were infested by spinneret-ablated (i.e., no BmFHD secretion) silkworms, and this increase resulted in the promotion of egg depositions by *Z. dolosa*. In addition to *Z. dolosa*, there are several other parasitoid flies that are known to respond to the induced indirect defense of mulberry plants, including *Blepharipa zebina* (Tachinidae)^22^, *Exorista sorbillans* (Tachinidae)^23^, and *E. japonica* (Tachinidae)^24^. Among them, *E. japonica* also uses GLVs such as (*Z*)-3-hexen-1-yl acetate and (*Z*)-3-hexen-1-ol as cues to find plants infested by herbivores^24^. Therefore, formation of GLVs by mulberry leaves under infestation could benefit the plants through attracting parasitoids more efficiently. Under this situation, herbivores that would have a countermeasure against the GLV-mediated induced indirect defense would have higher fitness.

Herbivore secretions have been reported to function as elicitors that are perceived by plants to induce HIPVs, but an inhibitory effect of herbivore secretions on plant volatile responses has also been reported^9,14–16^. For example, in tobacco (*Nicotiana tabacum*), feeding by *H. virescens* larvae with intact spinnerets induced lower amounts of some (eight out of 19 compounds) of the volatile compounds such as terpenoids, nicotine and one GLV [(*Z*)-3-hexen-1-yl tiglate], compared to feeding by caterpillars with ablated spinnerets^14^. Therefore, the ability of herbivore secretions to suppress HIPVs that might be attractive to the natural enemies of herbivores is likely one of countermeasures against plant indirect defense. However, it has not been clarified whether the suppression of HIPV formation by the secretion would affect performance of parasitoids. The factor in secretions that accounts for the suppression has not been identified. In our study, we showed that the secretion deposited via spinnerets of silkworm during feeding suppressed GLV formation by mulberry leaves and also showed that the suppression resulted in suppression of oviposition behavior of a parasitoid (*Zenillia dolosa*). We also identified a novel enzyme, BmFHD, as the factor in secretion responsible to the suppression of GLVs.

BmFHD catalyzes an unprecedented reaction to eliminate water from fatty acid hydroperoxide (HPO). Soybean LOX^25^ and rabbit reticulocyte LOX^26^ convert their main products, fatty acid HPOs, into their corresponding oxo-fatty acids. A fatty acid peroxide-metabolizing catalase in *Fusarium graminearum* catalyzes a similar reaction^27^. In the tubers of Jerusalem artichoke, *Helianthus tuberosus*, the oxidation of the hydroxide derived from fatty acid HPOs to carbonyl is catalyzed by a flavin dehydrogenase in an oxygen-dependent manner^28^. BmFHD has no similarity to LOXs, catalases, or flavin enzymes, and apparently needs no cofactor to complete its catalysis. Based on the unprecedented reaction catalyzed by BmFHD and the unprecedented primary structure of it, BmFHD is a novel enzyme. This enzyme had strict substrate specificity to 13(*S*)-HPOs of 18 carbon fatty acids. Such stereospecificity to the substrate would not be required for general antioxidant enzymes. Thus, it was less likely that BmFHD played a role as an antioxidant enzyme in silkworms. Rather, we infer that BmFHD is a specific enzyme for suppressing GLV productions at the fed edge. This inference was supported by the fact that *BmFHD* expression was high when silkworms were feeding mulberry leaves but low during the resting and wandering stages.

Several lepidopteran species have gene(s) homologous to BmFHD. It has been reported that caterpillar regurgitant of *Pieris rapae*, which has a BmFHD-like gene, suppressed formation of GLVs in *Arabidopsis* plants^16^. The ability to suppress GLV formation was also detected in silk gland extracts prepared from larvae of two Noctuidae species, *Mythimna separata* and *Spodoptera litura* (Supplemental Fig. 11). Accordingly, it is assumed that the ability to suppress GLV formation with BmFHD-like enzyme in the secretion is widespread among lepidopterans. In most lepidopteran larvae, the silk glands are specialized to produce prepupal cocoons^29^. Nevertheless, larvae spin silk for reasons other than cocoon making. For example, the presence of silk positively influenced the probability of a female of the two-spotted spider mite *Tetranychus urticae* to lay an egg^30^. Solitary lepidopteran larvae also leave silken thread on leaves. For example, diamondback moth (*Plutella xylostella*) neonates spin silk on cabbage leaf surfaces^31^. It was also reported that silk of the cabbage white butterfly larvae (*Pieris brassicae*, Pieridae) was left in a glass Petri dish in which larvae had been confined^32^. Here we added another reason to spinning silk by lepidopteran larvae as an effector suppressing induced indirect defense. Our study showed that genes homologous to BmFHD occurred in all Lepidopterans examined here, which implies that the countermeasure is widespread among lepidopteran species. A comprehensive search of *BmFHD-like* genes in Lepidopterans is required to extend this reasoning to lepidopteran species since there is a lack of information on the genes in other lepidopteran species. The function of *BmFHD-like* gene in a hymenopteran, *Athalia rosae* (Tenthredinidae) found here with slight homology to BmFHD should be also examined to know how and when the strategy to suppress GLV formation with the enzyme in secretions was acquired during the course of evolution of insects.

## Conclusions

We found a novel protein, BmFHD, in the secretions from a spinneret of a silkworm that suppressed the biosynthesis of GLVs in damaged cells of mulberry leaves by hampering one of steps [13*S*-HPOT to (*Z*)-3-hexenal] in GLV production pathway. Suppression of this pathway resulted in the reduced foraging efficacy of parasitoid flies, *Z. dolosa*. BmFHD is likely a countermeasure of silkworms against induced indirect defense with GLVs. This finding adds a further insight into plant-herbivore interactions at their contact site, where an herbivore secretion suppresses an induced indirect defense by a plant. GLVs are also involved in functions other than supporting the induced indirect defense, such as induction of plant direct defenses against herbivores and pathogens^5,6,33^. A further investigation is needed in order to clarify perspective of the ecological function of BmFHD,

## Methods

### Insects and plants

Silkworms of the Kinshu × Showa strain (wild-type) were purchased from Kougensha Co. Ltd (Nagano, Japan). Those of the N4 strain (wild-type) were obtained from and maintained at the University of Tokyo. The Kinshu × Showa strain was used for protein purification and subsequent analysis of the purified enzyme, while the N4 strain was used for analysis of gene expression. The neonates (200 to 300) hatched on the same day were collected and fed on mulberry leaves. The larvae that molted on the same day were randomly chosen for each experiment. For the spinneret-ablation experiment, five fifth instars were randomly chosen. For the RT-PCR, three fifth instars (for tissue-specificity) and three instars ranging from the second day of the fourth instar to the sixth day of the fifth instar (for temporal profiling) were randomly chosen from a population consisting of approximately fifty individuals.

Ablation of *B. mori* spinnerets was conducted to prevent secretion from silk glands. To ablate the spinneret, the head and ventral portion of one day-old fifth instars were fixed on a dish with adhesive tape. The spinnerets were cauterized by touching them briefly with a heat pen (at 240–270 °C, Towada Giken Co., Kanagawa, Japan).

The parasitoid fly *Zenillia dolosa* and its lepidopteran host *Mythimna separata* (Noctuidae) were obtained from a stock culture in the insect laboratory of Japan International Research Center for Agricultural Sciences, Tsukuba, Japan. Larvae of *M. separata* were reared on an artificial diet^34^ and *Z. dolosa* were reared on final (sixth) instars of *M. separata* as hosts^35^. Male and female flies (total 20–30 flies) were kept together for 10 to 20 days for mating after adult emergence for mating in a separate cage (23 cm length, 35 cm width, 20 cm height) with a sugar cube and water. All experiments were conducted at 25 °C under a photoperiod of 16 h light to 8 h dark and 60% relative humidity.

Mulberry trees (*Morus alba* L. cv. Shin-Ichinose) grown from cuttings produced in 2002 in a common garden at the University of Tokyo (35°42′N, 139°45′E) were used throughout this study. For the experiments that examined the effect of the silk-gland extract on GLV formation, the mulberry trees (*M. alba* L. cv. Senshin) that were grown from cuttings obtained in March 2012 in a common garden at Yamaguchi University (34°15′N, 131°48′E) were used.

### Effect of spinneret secretions on mulberry leaf volatiles

Young, fully expanded mulberry leaves were cut at the base of the petioles and the petioles were immediately inserted in vials (7 mL) filled with distilled water. Each leaf and vial was enclosed in a cylindrical plastic case (490 cm^3^) (10.9 cm i.d., 5.3 cm height), and two caterpillars with either intact or ablated spinnerets were allowed to feed on the leaves for 6 h at 25 °C under two fluorescent lights (FHF32EX-N-K, 32 W, Hitachi, Ltd., Tokyo, Japan). The leaf without caterpillars was also treated under the same conditions. The distance between the leaves and the fluorescent light was 18 cm (169 µmol m^−2^ s^−1^ at the surface of leaves). The leaf area (cm^2^) consumed by the larvae was determined using Image J software (imagej.nih.gov/ij/) with photos taken before and after the feeding period.

To collect volatiles induced by larval feeding, a MonoTrap cartridge (silica monolith matrix coated with octadecyl silyl group and activated carbon) (RCC18, GL Sciences Inc., Tokyo, Japan) was attached to the cover of the plastic case from the beginning to the end of the feeding period (for 6 h). Volatiles were eluted from each cartridge with 200 µL of dichloromethane (including 5 ng mL^−1^ of vanillin as an internal standard) and analyzed by gas chromatography (GC)-mass spectrometry (MS) (GCMS QP2010SE, Shimadzu, Kyoto, Japan) using a DB-WAX capillary column (30 m × 0.25 mm, 0.25 µm film thickness, Agilent, Santa Clara, CA, USA). The injection temperature was 250 °C (splitless injection mode; sampling time, 1 min). The GC oven program was maintained at an initial temperature of 40 °C (held for 5 min), followed by a ramp of 5.0 °C min^−1^ to a final temperature of 200 °C (held for 2 min). The electron ionization mode with an ionization voltage of 70 eV was used, and the *m/z* from 40 to 400 was recorded. Peaks for (*Z*)-β-ocimene, (*E*)-β-ocimene [≥90% (mixture of isomers), Sigma-Aldrich, St. Louis, MO, USA], (*Z*)-3-hexen-1-yl acetate (≥97.0%, Tokyo Chemical Industry, Tokyo, Japan), and (*Z*)-3-hexen-1-ol (≥97.0%, Wako Pure Chemicals, Osaka, Japan) were assigned based on comparison of mass spectra and retention times with authentic compounds. Other peaks were assigned based on the comparison of mass spectrum using the NIST27 and NIST147 GC-MS libraries (GCMS Solution, Shimadzu). For (*Z*)-3-hexen-1-yl acetate and (*Z*)-3-hexen-1-ol, calibration curves were constructed with the authentic compounds.

### Behavioral response of *Z. dolosa* female flies to mulberry leaf odorants

We collected volatiles from the headspace of mulberry leaves infested by normal silkworms or the spinneret-ablated silkworms with MonoTrap as odor samples. One mulberry leaf was enclosed in a plastic case (490 cm^3^), and three silkworms with either intact or ablated spinnerets were allowed to feed on the leaves for 2 h at 25 °C under two fluorescent lights. The headspace volatiles were collected for 2 h with a MonoTrap cartridge as described for the experiment to examine the effect of spinneret secretion on mulberry leaf volatiles. Volatiles were eluted from the cartridge with 200 µL of dichloromethane and stored at −30 °C until use.

Behavioral experiments involving *Z. dolosa* females were conducted in a glass container (3 cm i.d. × 4.5 cm). Mated females were transferred into the glass container containing a sugar cube and kept for 10–15 min. A droplet (5 µL) of solvent (dichloromethane) or collected volatiles in the solvent was impregnated into a filter paper disc (0.6 cm diameter) on the glass petri dish. The filter paper was left outside of the container for 30–40 sec to allow the dichloromethane to evaporate. The filter paper and one mated female fly were then introduced together into the glass container. We observed the oviposition behavior of the fly for 5 min. The bioassay was carried out on three different days using a total of 69 *Z. dolosa* females (23 for each odor source or dichloromethane as control). The odor solution, filter paper, and glass container were replaced for each replicate of the bioassay.

### Volatile analysis of ruptured mulberry leaves treated with silk gland extract

To prepare the silk gland extract, the fifth instars were dissected on ice, and silk glands were divided into three parts: (1) the anterior silk glands plus the anterior part of the middle silk glands (ASG + MSG-A), (2) the middle to posterior parts of the middle silk glands [MSG(M-P)], and (3) the posterior silk glands (PSG). Each section was ground to a powder in liquid nitrogen. A portion (0.2 g) of the powder was homogenized with 1.5 mL of extraction buffer (20 mM MES-NaOH, pH 5.6) by using a beads-type cell disintegrator (Micro smash, MS-100R, Tomy Seiko, Tokyo, Japan) with seven steel beads (3 mm i.d.). The homogenate was centrifuged at 14,000 rpm at 4 °C for 20 min, and the supernatant was used for the experiment. Fresh mulberry leaves (cv. Senshin) were snap-frozen and ground to a powder in liquid nitrogen, and a portion (0.2 g) of the leaf-powder was homogenized by using the same technique as detailed above, with 100 µL of the extraction buffer containing a given volume of silk gland extract. After homogenization, the homogenates were incubated at 25 °C for 20 min, and then mixed with 1 mL of methyl *tert*-butyl ether including 1 µg mL^−1^ of nonanyl acetate as an internal standard by using a beads-type cell disintegrator. The mixture was centrifuged at 14,000 rpm at 4 °C for 20 min, and the upper organic phase was served for GC-MS analysis with GC-MS QP-5050 (Shimadzu) equipped with a DB-WAX capillary column (30 m × 0.25 mm, 0.25 µm film thickness, Agilent). The injection temperature was 240 °C (split injection mode, split ratio: 1:10). The GC oven program was an initial temperature of 40 °C (held for 5 min), followed by a ramp of 7.5 °C min^−1^ to a temperature of 150 °C, followed by a ramp of 10 °C min^−1^ to a final temperature of 200 °C (held for 2 min). The electron ionization mode with ionization voltage at 70 eV was used, and the *m/z* from 40 to 350 was recorded. (*E*)-2-Hexenal (>97%, Wako Pure Chemicals, Osaka, Japan), (*E*)-2-hexen-1-ol (95%, Tokyo Chemical Industry, Tokyo, Japan), (*Z*)-2-penten-1-ol (97%, Alfa Aesar, Lancashire, UK), and 1-penten-3-ol (>98%, Alfa Aesar) were used to identify the compounds by comparing the retention indices and MSs. Generally, in intact plant leaves, the amounts of GLVs are low, but within seconds after disruption of plant tissues, linolenic acid is oxygenated to form linolenic acid 13-HPO, which is subsequently cleaved by HPL to form (*Z*)-3-hexenal^5^. A portion of (*Z*)-3-hexenal diffuses out to the neighboring intact tissues, where it is reduced to form (*Z*)-3-hexen-1-ol and further acetylated to form (*Z*)-3-hexen-1-yl acetate^36^, and therefore, the alcohol and acetate were the major GLVs detected in headspace (Fig. 1). On the other hand, (*Z*)-3-hexenal stayed in the disrupted tissues is isomerized to form (*E*)-2-hexenal in some plant species^37,38^. Thus, (*E*)-2-hexenal and (*E*)-2-hexen-1-ol were major volatiles in the disrupted mulberry leaves.

### Activity assay

13(*S*)-HPOs of linoleic and linolenic acid were prepared with partially purified soybean seed LOX-1 as reported previously^39^. The HPOs were purified to remove fatty acids with silica gel chromatography (Wakogel C-300, Wako Pure Chemicals, Osaka, Japan) with the solvent system of hexane/ether. Reduction of the HPOs to corresponding hydroxides was carried out by the addition of a large excess of triphenylphosphine (Wako Pure Chemicals). Conversion of the HPOs was monitored by following absorption at 234 nm with a spectrophotometer. In a typical assay, 2 µL of 10 mM 13*S*-HPOT in ethanol was added into 1 mL of 50 mM sodium borate buffer (pH 9.0) containing an appropriate amount of enzyme solution. Formation of the corresponding oxo-fatty acid was monitored by reading absorption at 280 nm. After the reaction, the mixture was acidified to pH 4.0 with perchloric acid, and the products were extracted with ether. The ether extract was dried with nitrogen gas. The resulting residue was dissolved in 100 µL of hexane/2-propanol/acetic acid (98:2:0.05 by volume) for HPLC analysis as follows.

### Substrate and product analyses

The mixture of isomers of HPOs of linoleic acid was prepared by autooxidation by incubating linoleic acid (1 g, >99%, Sigma) at 40 °C for 55 h^40^. 13-OTE and 9-OTE were prepared by reaction of α-linolenic acid (>99%, Sigma) with crude LOX prepared from soybean seeds^41^. The reaction was carried out in 20 µL of 50 mM α-linolenic acid in 978 µL of 0.2 M sodium phosphate buffer (pH 6.1) at 25 °C. After confirming a substantial increase of absorption at 280 nm, the products were extracted with ether after adjusting the pH to 4.0 with perchloric acid. The ether phase was dried with nitrogen gas, and the resulting residues were dissolved in 100 µL of hexane/2-propanol/acetic acid (98:2:0.05 by volume). The straight phase HPLC was conducted with Shimadzu LC-20AD system equipped with a ZORBAX SIL column (4.6 × 250 mm, Agilent). The positional and geometrical isomers of HPOs and ketones of linoleic acid and α-linolenic acid were separated with hexane/2-propanol/acetic acid (98:2:0.05 by volume) at a flow rate of 0.75 mL min^−1^ with a monitoring absorption of 220–350 nm using a diode array detector (Shimadzu SPD-M20A). 9-Oxo-10*E*,12*Z*-octadecadienoic acid (>98%) and 13-oxo-9*Z*,11*E*-octadecadienoic acid (98%) were purchased from Cayman Chemical (Ann Arbor, MI). Further confirmation of 13-OTE was accomplished with a HPLC-MS/MS system [3200Q-TRAP (AB Sciex, Framingham, MA) equipped with a Prominence UFLC (Shimadzu)]^20,41,42^. 13-KOT was separated on a Mightysil RP18 column (2 × 150 mm, Kanto Chemical, Tokyo, Japan) with a binary gradient consisting of water/formic acid (100:0.1, v/v, solvent A) and acetonitrile/formic acid (100:0.1, v/v, solvent B). The run consisted of a linear increase from 60%B to 80%B over 25 min (flow rate, 0.2 ml min^−1^). 13-OTA was detected with absorption at 280 nm, and by MS/MS either with enhanced-MS mode or enhanced-product ion mode using electrospray ionization in the negative ion mode [ion spray voltage, −2700 V, nitrogen as both the curtain gas (set to 30 arbitrary units) and collision gas (set to “high”); collision energy, −10 V; scan range, m/z 50–800; scan speed, 4000 Da s^−1^; declustering potential, −25 V]. For enhanced-product ion mode, the molecular ion of *m/z* 291.2 (corresponding to [M − H]^−^) was selected, and fragmented with the collision energy of −45 V. For determination of the stereochemistry of 13-hydroperoxy-(9*Z*,11*E*)-octadecadienoic acid, the peak that corresponded to this HPO with the straight-phase HPLC was fractionated. Each enantiomer was separated with a Chiralcel OD-H column (4.6 × 250 mm, Daicel Chemical Industries, Osaka, Japan) with a solvent system of hexane/2-propanol/acetic acid (98/2.0/0.1, v/v) at a flow rate of 0.75 mL min^−1 ^^43^. Under the HPLC conditions, the 13*R*-isomer elutes earlier than the 13*S*-isomer.

### Purification of BmFHD from silk glands

Middle silk glands (MSG-A) collected from fifth instar silkworms were ground to a powder in liquid nitrogen. A portion (0.8 g) of the powder was homogenized with 8 mL of extraction buffer (50 mM Tris-HCl, pH 9.0) by using a Potter-Elvehjem tissue grinder. The homogenate was centrifuged at 20,000 × g at 4 °C for 20 min, and the supernatant was dialyzed with dialysis tubing of molecular weight cut-off 12–14 kDa (Spectrum Laboratories, Inc., Rancho Dominguez, CA). Dialyzed solution was applied to a Cellufine Q-500 column (1 × 10 cm, JNC Co., Tokyo, Japan) pre-equilibrated with the extraction buffer. The column was eluted with a linear KCl gradient (0–1.0 M) with the extraction buffer at a flow rate of 60 mL min^−1^. Fractions with high activity were combined and applied to a HiTrap Butyl HP (1 mL, GE Healthcare Life Science Ltd., Little Chalfont, UK). The activity was eluted by the extraction buffer including 30% (w/v) glycerol. After purification, the protein band of 32 kDa on SDS-PAGE gel was cut off, and served to in-gel trypsin digestion, and the trypsin-digested peptides were analyzed by HPLC-MS/MS to identify the protein. LC-MS/MS analysis was performed by a linear ion trap time-of-flight mass spectrometer (LIT–TOF MS), NanoFrontier eLD (Hitachi High-Technologies Co., Tokyo, Japan) coupled to a nanoflow HPLC, NanoFrontier nLC (Hitachi High-Technologies Corporation). The trypsin-digested peptides were separated using the Capillary EX-Nano column (0.05 × 150 mm, GL Science, Tokyo, Japan) and eluted with a linear gradient from 2 to 40% solvent B in 60 min at a flow rate of 200 nL min^−1^. Solvent A was 2% acetonitrile and 0.1% formic acid, and solvent B was 98% acetonitrile and 0.1% formic acid. The eluent was ionized with a nano-electrospray ionization source equipped with an uncoated SilicaTip (New Objective, Woburn, MA, USA) and analyzed with a LIT–TOF MS. Mass spectra were obtained in positive ion mode at scan mass range of *m/z* 200–2000. MS/MS spectra were generated by collision-induced dissociation in the linear ion trap. To identify the protein, the MS and MS/MS data were converted to an MGF file using the NanoFrontier eLD Data Processing software (Hitachi High-Technologies Co.) and analyzed with the MASCOT MS/MS Ions Search (http://www.matrixscience.com) using the following parameters: database, NCBInr; enzyme, trypsin; missed cleavages, 3; taxonomy, all entries; fixed modifications, carbamidomethyl (C); variable modifications, i.e. oxidation (HW) and oxidation (M); peptide tolerance, 0.2 Da; MS/MS tolerance, 0.2 Da; Peptide charge, 1+, 2+, and 3+; and Instrument, ESI-TRAP.

### cDNA cloning

Total RNA was extracted from the middle silk glands of the fifth instar of the silkworms (Kinshu × Showa strain) using TRIzol reagent (Invitrogen, Carlsbad, CA, USA). DNA was degraded using DNA-free Kit (Ambion, Thermo Fischer Scientific, Waltham, MA, USA). cDNA was synthesized with SuperScript VILO cDNA Synthesis Kit (Invitrogen), then BmFHD cDNA was PCR-amplified by using primers (SF001 and SF002) designed based on the sequence of the gene (LOC101739721, acc. no. XM_004923484.2) from GenBank (http://www.ncbi.nih.gov/GenBank/). The primer sets that were used in this study are listed in Supplemental Table 2. RT-PCR was conducted using the SuperScript VILO cDNA Synthesis Kit (Invitrogen) under the following conditions: 94 °C for 2 min, 30 cycles at 94 °C for 15 sec, 58 °C for 30 sec, and 68 °C for 60 sec, followed by 72 °C for 5 min. The PCR product was cloned into pENTER/D-TOPO vector (Invitrogen), and its sequence was determined. For secretory production of recombinant BmFHD in the insect cell/baculovirus expression system, the *att*L-flanked PCR-amplified cDNA in the entry vector pENTER/ D-TOPO was transferred into the destination vector pBm14-GW, a modified version of the transfer vector pBm4^44^ containing the *att*R-flanked *ccdB* gene cassette between 5′ and 3′ flanking regions of the Bombyx mori nucleopolyhedrovirus (BmNPV) polyhedrin gene, by using Gateway LR Clonase Enzyme Mix (Invitrogen). The resulting pBmGW/FHD was transfected with BmNPV DNA into BmN4 cells derived from *B. mori*, and recombinant viruses containing the BmFHD cDNA were purified from supernatant of the transfected cells by two cycles of plaque purification. Secretion of functional BmFHD in the culture supernatant of the recombinant virus-infected BmN4 cells was confirmed by activity assay as described above.

### Phylogenetic analysis

The deduced amino acid sequence of BmFHD (DDBJ accession number; LC259005) was used as the query in a TBLASTN search against the scaffold databases for *Papilio xuthus, Heliconius melpomene, Amyelis transitella, Plutella xylostella, Chilo suppressalis, Spodoptera litura*, and *Pieris rapae*. All the sequences with substantial similarity were chosen, and those deposited as protein sequences were further selected to construct the phylogenetic tree. TBLASTN search was also performed against the NCBI genome (http://www.ncbi.nlm.gov/genome/) (for *Athalia rosae, Drosophila melanogaster, Anopheles gambiae, Acyrthosiphon pisum*, and *Nilaparvata lugens*). Multiple alignment of amino acid sequences were performed using ClustalW alignment software (Supplemental Fig. 10). Phylogenetic analyses were performed using the neighbor-joining (NJ) method in MEGA5^45^. The NJ method was performed based on a p-distance model with pairwise deletion of gaps/missing data treatment with a bootstrap test of 1,000 replications.

### RT-PCR

For a tissue-specific expression profile of BmFHD, the fifth instars (third day, N4 strain) were dissected on ice. The head, anterior silk glands (ASG), anterior part of the middle silk glands (MSG-A), middle-to-posterior parts of middle silk glands [MSG(M-P)], posterior silk glands (PSG), salivary glands (SAG), midgut (MGUT), small gut plus colon plus rectum (SGUT + C + R), Malpighian tube (MT), and fat body plus epidermis (FB + E) were isolated. For stage-specific expression profiles of BmFHD in the anterior part of the middle silk glands (MSG-A), samples of the N4 strain on each day were collected from the second day of the fourth instar to the sixth day of the fifth instar. Total RNA was isolated using TRIzol reagent. First strand cDNA was prepared using an oligo (dT) primer and avian myeloblastosis virus reverse transcriptase from a TaKaRa RNA PCR kit (Takara Bio Inc., Otsu, Japan). To quantify the amount of mRNA, quantitative RT-PCR was performed with a KAPA SYBR FAST qPCR kit (Kapa Biosystems, Boston, MA, USA) and an ABI StepOne Plus Real-Time PCR System (Applied Biosystems, Thermo Fisher Scientific). The primers used for quantitative RT-PCR are listed in Supplemental Table 2. The transcript level of *Bombyx mori ribosomal protein 49* (*Bmrp49)* was used for normalization.

### Immunoblot

Tissues of fifth instars (N4 strain, head; anterior silk glands, ASG; anterior parts of middle silk glands, MSG-A; middle parts of middle silk glands, MSG-M; posterior parts of middle silk glands, MSG-P; posterior silk glands, PSG) were homogenized with extraction buffer (30 mM sodium phosphate, pH 6.6). The spinneret secretion was suspended with the extraction buffer. The regurgitant that was collected by inserting a capillary into the mouth of the larvae was used without dilution. Samples were centrifuged at 14,000 rpm for 20 min at 4 °C to collect supernatant. The protein content was determined by using Coomassie Plus Protein Assay Reagent (Thermo Fischer Scientific). A portion containing 1 µg of protein was subjected to SDS-PAGE, and proteins were electro-transferred onto polyvinylidene difluoride membrane (Merck Millipore Ltd., Billerica, MA, USA). Polyclonal antibody against a synthetic peptide, CKLSNTYKWFEKPSKAA derived from the internal amino acid sequence of BmFHD, was raised with rabbits by using the custom antibody production services (Sigma-Aldrich). BmFHD was detected with 1:1000 diluted primary antibody and 1:2000 diluted secondary antibody conjugated with horseradish peroxidase (Invitrogen), and color development with Immobilon Western Chemiluminescent HRP substrate (Millipore Corporation). The band intensity was quantified with ImageJ software (https://imagej.nih.gov/ij/). In order to determine the amount of BmFHD protein deposited on the surface of mulberry leaves that were fed on by silkworms, the wounded edge (ca 0.3 cm width) on the mulberry leaf, made by a silkworm over a 2-h duration, was cut out and immersed in an extraction solution (1 mL) consisting of 50 mM Tris-HCl (pH 9.0) and 0.9% (w/v) sodium dodecyl sulfate for 1 h. The solution (950 µL) was cleared by centrifugation at 14,000 rpm for 20 min at 25 °C, and concentrated with ultrafiltration to 100 µL. A portion (15 µL) of the solution was served to the immunoblot analysis. Known amounts of purified BmFHD were loaded onto the gel and processed in parallel with extracts from the wounded edges. After densitometric analysis of the band intensity corresponding to BmFHD with ImageJ, the amount of BmFHD on the wounded edges was estimated.

### Statistical analysis

We checked all numeric data of treatment groups graphically and statistically (Shapiro-Wilk test and Bartlett test) for normality and homoscedasticity using JMP software (version 10.0.2; SAS Institute, Cary, NC, USA). For the silkworm-induced volatile analysis (Fig. 1C), statistical analyses were conducted using Welch’s *t*-test in JMP. The leaf areas consumed by silkworms (Supplemental Fig. 2) was analyzed using *t*-test in JMP. For multiple comparisons of oviposition rates of *Z. dolosa* females, pairwise tests were conducted with Fisher’s exact test in JMP followed by Holm’s *P*-value adjustment (Fig. 2B). The number of eggs laid by *Z. dolosa* females was analyzed with Steel-Dwass test (Fig. 2C). To analyze the effects of the amount of the silk gland extract and BmFHD purified from the silk gland (Fig. 3B, Supplemental Fig. 3), pairwise tests were conducted with generalized linear model (GLM) assuming a normal distribution and identity-link in JMP followed by Holm’s *P*-value adjustment. To analyze the effect of silkworm tissue on the expression profile of *BmFHD* mRNA (Fig. 4A), statistical analysis was conducted using a generalized linear mixed model (GLMM) with a normal distribution and identity-link using the function lmer in the package lme4 version 1.1.7^46^ in R version 3.1.1^47^. The individual silkworm was the random effect in the models and the significance was evaluated using a likelihood ratio test comparing models with or without the effect of the tissue. All possible pairwise combinations of tissues were compared by GLMM. The *P*-values of the likelihood ratio tests were adjusted by Holm’s method. For multiple comparisons of temporal expression profile of *BmFHD* mRNA (Fig. 4B), pairwise tests were conducted using GLM with a normal distribution and identity-link in the JMP followed by Holm’s *P*-value adjustment. All of the data on the formation of GLVs, expression profile of BmFHD mRNA in each tissue and temporal expression profile of *BmFHD* mRNA were Box-Cox transformed using JMP before conducting statistical analyses. All data on the formation of GLVs were increased by a value of 1 before being Box-Cox transformed to avoid having values of 0.

### Availability of data and materials

The datasets used and/or analyzed during the current study are available from the corresponding author on reasonable request. The sequence reported in this paper has been deposited in the DNA Data Bank of Japan, http://www.ddbj.nig.ac.jp/ (accession no. LC259005).

## Electronic supplementary material

Supplementary Information

Supplemental Movie 1
