# Supplementary material for: Silkworms suppress the release of green leaf volatiles by mulberry leaves with an enzyme from their spinnerets
Source: Sci Rep. 2018 Aug 9;8:11942. doi: 10.1038/s41598-018-30328-6 (PMC6085349; doi:10.1038/s41598-018-30328-6)
Supplement: Supplementary file 1 — Supplementary Information [file 41598_2018_30328_MOESM1_ESM.pdf]

Supplementary Information for

Silkworms suppress the release of green leaf volatiles by mulberry leaves with an enzyme from their spinnerets

Hiroki Takai[1,2], Rika Ozawa[3], Junji Takabayashi[3], Saki Fujii[4], Kiriko Arai[1], Ryoko T. Ichiki[5], Takao Koeduka[1], Hideo Dohra[6], Toshiyuki Ohnishi[6,7], Sakura Taketazu[8], Jun Kobayashi[1], Yooichi Kainoh[9], Satoshi Nakamura[5], Takeshi Fujii[10], Yukio Ishikawa[10], Takashi Kiuchi[2], Susumu Katsuma[2], Masayoshi Uefune[11], Toru Shimada[2], Kenji Matsui[1]*

[1] Division of Agricultural Sciences, Graduate School of Sciences and Technology for Innovation, Yamaguchi University, Yamaguchi 753-8515, Japan
[2] Laboratory of Insect Genetics and Bioscience, Graduate School of Agricultural and Life Sciences, The University of Tokyo, Tokyo 113-8657, Japan
[3] Center for Ecological Research, Kyoto University, Otsu, Shiga 520-2113, Japan
[4] Department of Biological Chemistry, Faculty of Agriculture, Yamaguchi University, Yamaguchi 753-8515, Japan
[5] Japan International Research Centre for Agricultural Sciences, Tsukuba, Ibaraki 305-0851, Japan
[6] Research Institute of Green Science and Technology, Shizuoka University, Shizuoka 422–8529, Japan
[7] College of Agriculture, Academic Institute, Shizuoka University, Shizuoka 422–8529, Japan
[8] Department of Biological and Environmental Sciences, Faculty of Agriculture, Yamaguchi University, Yamaguchi 753-8515, Japan
[9] Faculty of Life and Environmental Sciences, University of Tsukuba, Tsukuba, Ibaraki 305-8577, Japan
[10] Laboratory of Applied Entomology, Graduate School of Agricultural and Life Sciences, The University of Tokyo, Tokyo 113-8657, Japan
[11] Department of Agrobiological Resources, Faculty of Agriculture, Meijo University, Nagoya, Aichi 468-8502, Japan.

***Correspondence author:**
Kenji Matsui: matsui@yamaguchi-u.ac.jp

Supplementary Table 1. Summary of purification of *Bombyx mori* fatty acid hydroperoxide dehydratase (BmFHD) from middle silk glands.

Purification steps	Total protein (mg)	Specific activity (mkat mg^{-1})	Total enzyme activity (mkat)	Purification (fold)	Yield (%)
Crude enzyme	9.58	0.17	1.60	1.0	100.0
Dialysis	9.59	0.16	1.53	0.9	95.6
Cellufine Q-500	1.94	0.47	0.92	2.8	57.5
HiTrap Butyl HP (Wash)	0.30	2.43	0.73	14.3	45.6
HiTrap Butyl HP (30% of glycerol)	0.0337	8.91	0.30	52.4	18.8

Supplementary Table 2. Primers used in this study.

Name	Sequence (5'-3' end)	Object
SF001	CACCATGAAGGCTTTGGCAGCTGTACTATTG	RT-PCR for XM_004923484.2
SF002	TCACTGCCACAGGCAACTGACCAACCATGG	RT-PCR for XM_004923484.2
BmFHD_F	TCCAAGGTCACTGGAAGAGGT	qRT-PCR for *BmFHD*
BmFHD_R	TACAAGGCGAGCGAAGGAA	qRT-PCR for *BmFHD*
rp49_RF2	CCCAACATTGGTTACGGTTC	qRT-PCR for *rp49*
rp49_RR2	GCTCTTTCCACGATCAGCTT	qRT-PCR for *rp49*

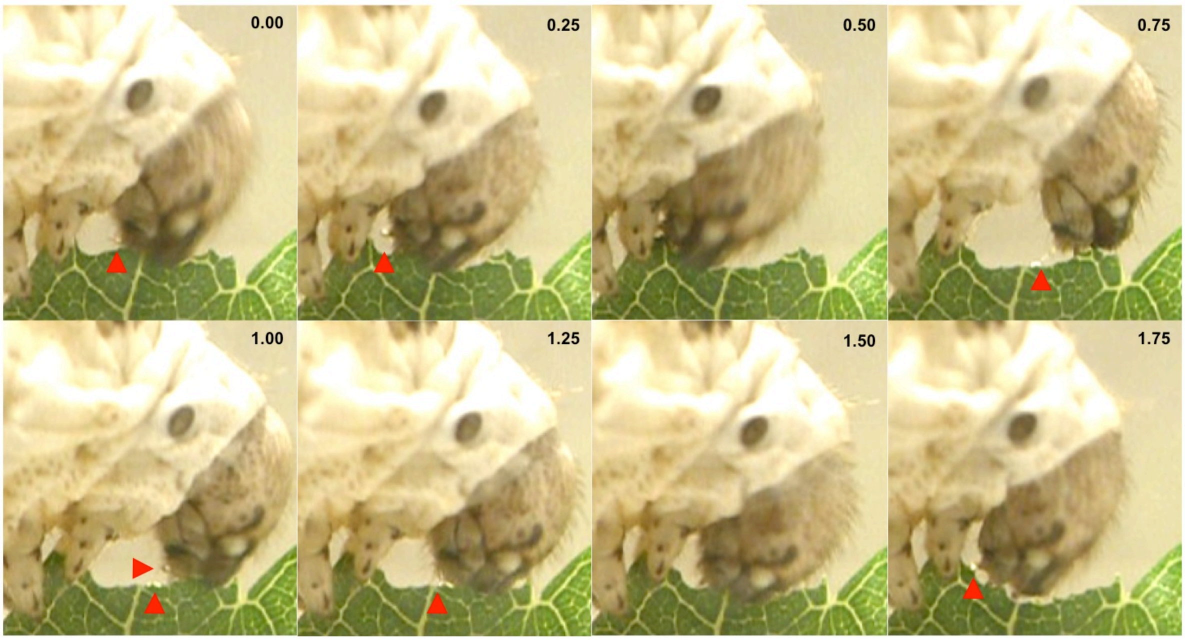

Supplementary Fig. 1. Photos captured a silkworm, *Bombyx mori,* feeding on a mulberry leaf, *Morus alba*. Before starting a new bite, the silkworm secreted a droplet (at 0 sec, arrow head) that attached to a section of the damaged leaf edge (at 0.50 sec). Thereafter, the silkworm re-oriented its head for another bite and as a result, it made a thread (at 0.75 sec, arrow head).

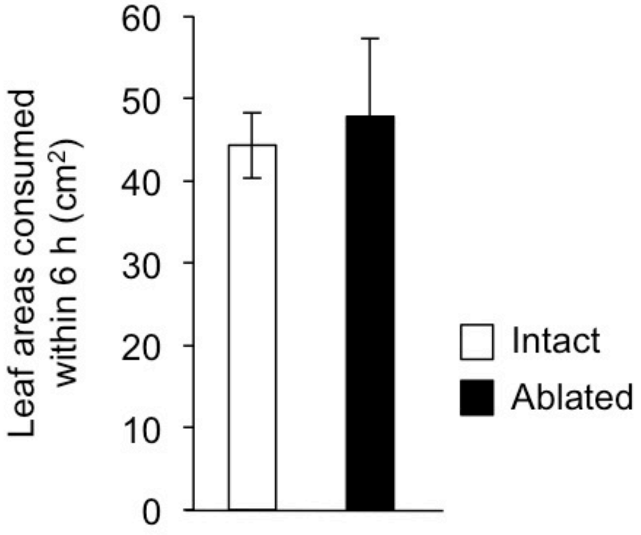

Supplementary Fig. 2. Leaf area consumed by silkworms. Leaf areas consumed after 6 h by silkworms with spinnerets (white) or without spinnerets (black) are shown. The means are shown with SE (n = 5). The leaf areas were not significantly different (t-test, P = 0.74).

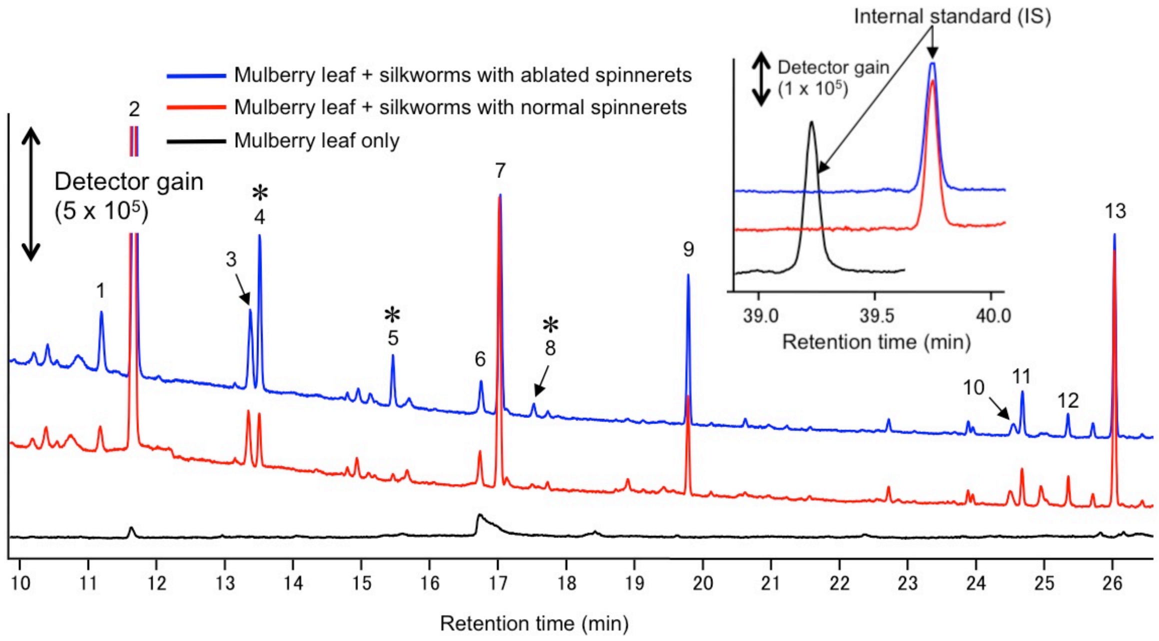

Compound	Retention time (min)
1 : (*Z*)-β-ocimene	1 : 11.2
2 : (*E*)-β-ociemene	2 : 11.6
3 : geranyl nitrile	3 : 13.3
4 : (*Z*)-3-hexen-1-yl acetate *	4 : 13.5
5 : (*Z*)-3-hexen-1-ol *	5 : 15.5
6 : unknown monoterpene 1	6 : 16.8
7 : unknown monoterpene 2	7 : 17.0
8 : (*Z*)-3-hexen-1-yl butanoate *	8 : 17.5
9 : linalool	9 : 19.8
10 : α-farnesene	10 : 24.5
11 : methyl salicylate	11 : 24.7
12 : 2-methyl-3-buten-2-ol	12 : 25.4
13 : (*Z*)-*p*-menth-2,8-dien-1-ol	13 : 26.0
IS : Vanillin	IS : 39.7

Supplementary Fig. 3.

Representative chromatograms of volatiles collected from mulberry leaves cut at the petiole (black trace), those infested with silkworms with normal spinnerets (red trace), and those infested with silkworms with ablated spinnerets (blue trace). Peaks of internal standard are shown in the inset. The compound name and retention time for each peak number is also shown.

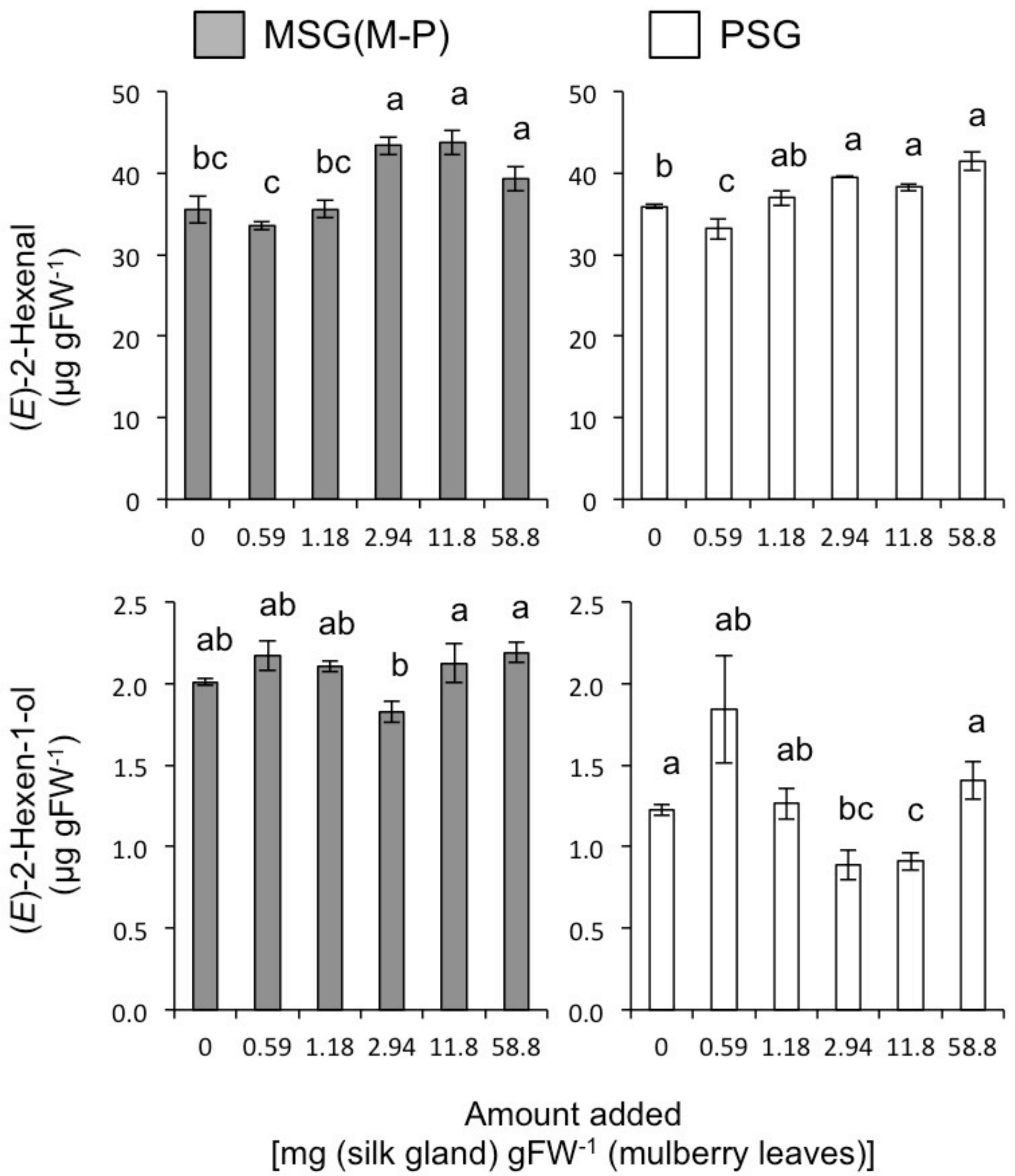

Supplementary Fig. 4. The middle to posterior parts of silk gland extract showed little effect on formation of green leaf volatiles. Green leaf volatile formation in the presence of silk gland extract was analyzed as shown in Fig. 3b. The amounts of (*E*)-2-hexenal and (*E*)-2-hexen-1-ol formed in the absence or presence of the silk gland extract that was obtained from the middle-to-posterior parts of the middle silk gland [MSG(M-P)] and the posterior silk gland (PSG). Averages with error bars (SE, $n = 3$, technical replicate) are shown. The lowest amount of the silk gland extract (0.59 mg) corresponded to 0.0047 [MSG(M-P)] or 0.059 (PSG) equivalent of that derived from one silkworm. Different letters above the bars indicate significant differences among treatments for each extract ($P < 0.05$, GLM following Holm's *P*-value adjustment).

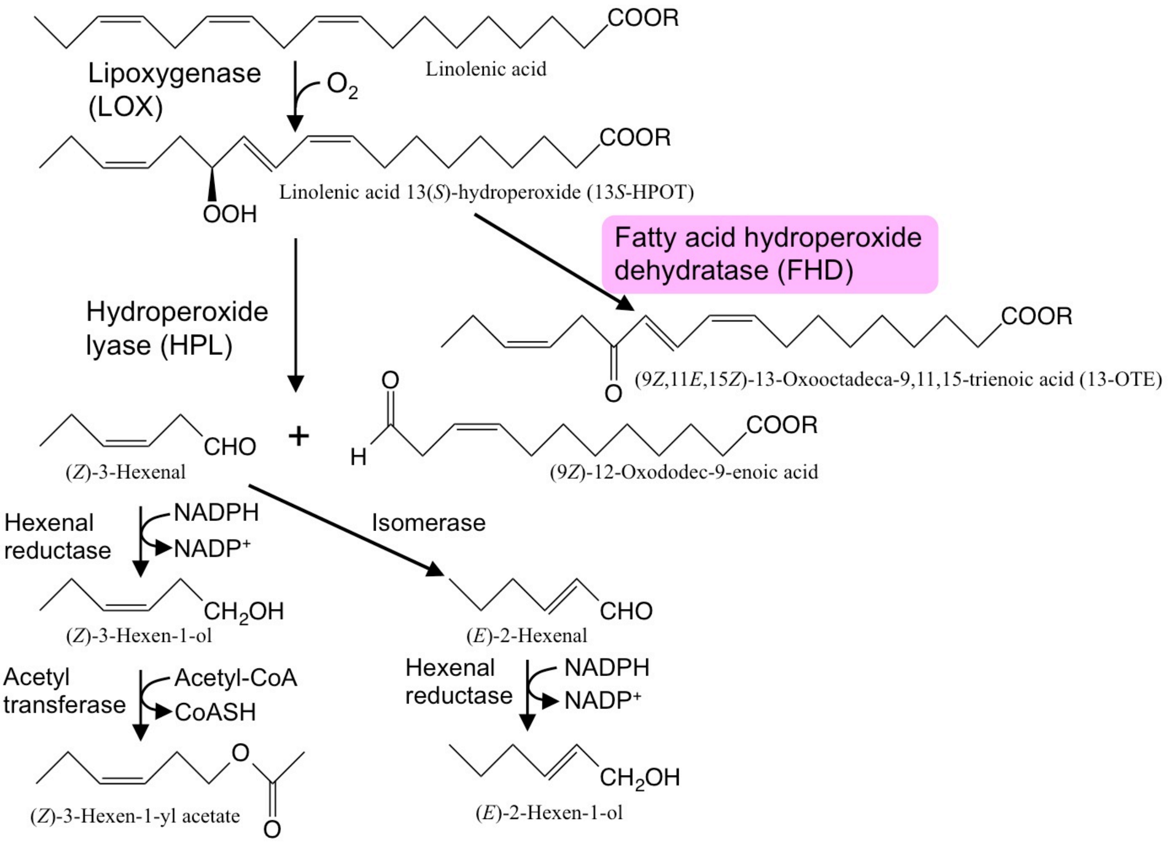

Supplementary Fig. 5. Biosynthetic pathway to form green leaf volatiles from linolenic acid. In the pathway, lipoxygenase (LOX) adds dioxygen at position 13 of linolenic acid to produce linolenic acid 13(*S*)-hydroperoxide (13*S*-HPOT). The hydroperoxide is cleaved by hydroperoxide lyase (HPL) at the C12–C13 bond to produce (*Z*)-3-hexenal, which can be reduced to form (*Z*)-3-hexen-1-ol. A portion of (*Z*)-3-hexen-1-ol is further converted to (*Z*)-3-hexen-1-yl acetate. In some plants, (*Z*)-3-hexenal is converted to (*E*)-2-hexenal spontaneously or enzymatically, and further reduced. The reduction and acetylation needs NADPH and acetyl-CoA. Fatty acid hydroperoxide dehydratase (FHD) convert 13*S*-HPOT to (9*Z*,11*E*,15*Z*)-13-oxooctadeca-9,11,15-trienoic acid (13-OTE).

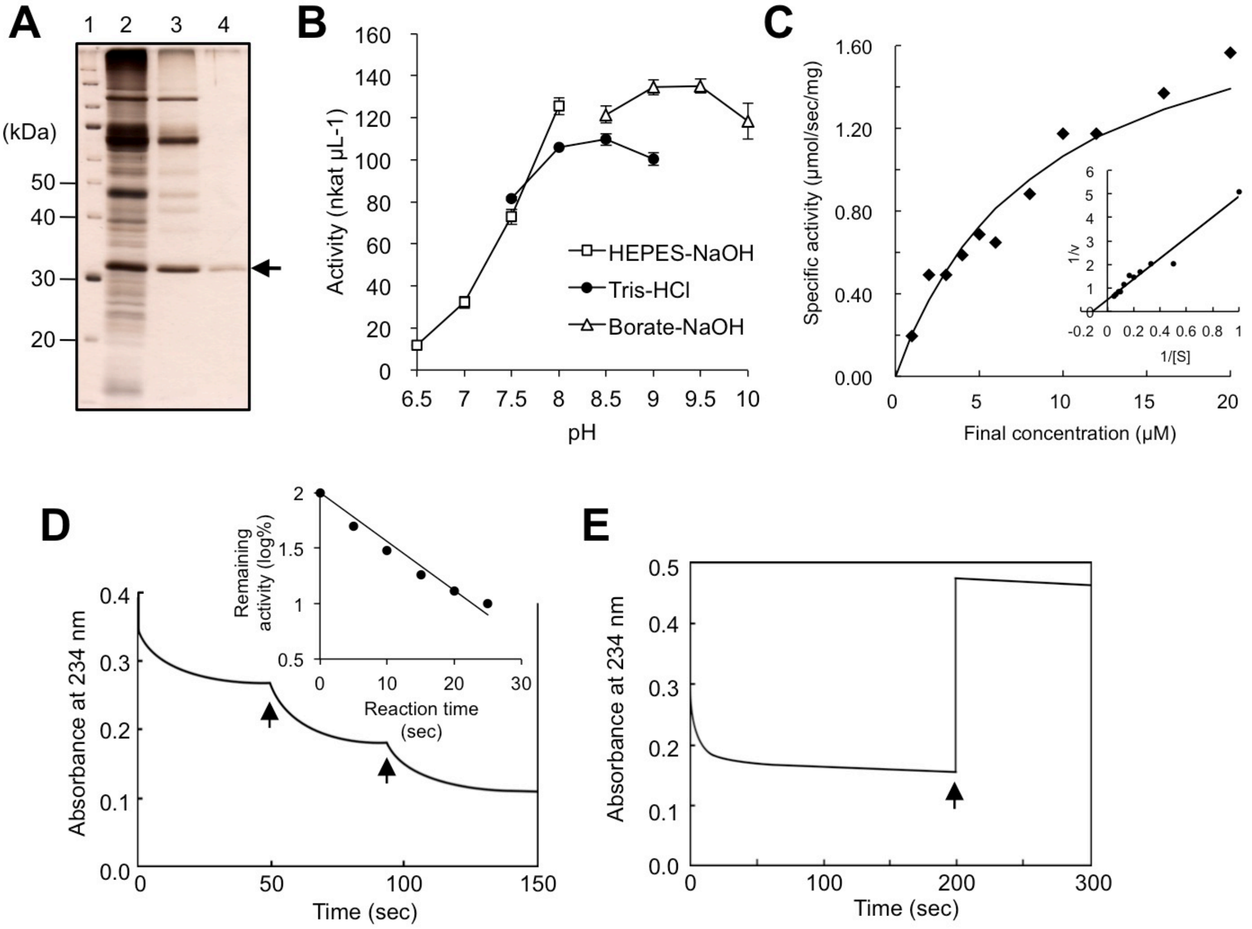

Supplementary Fig. 6. Properties of purified enzyme. (A) Protein profiles of each fraction obtained during purification of BmFHD from middle silk glands. Lane 1: molecular mass markers; 2: crude enzyme solution; 3: the anion exchange chromatography (Cellufine Q-500) fraction; 4: the hydrophobic interaction chromatography (HiTrap Butyl HP) fraction. The band corresponding to BmFHD is indicated with an arrow. **(**B**)** pH-activity profile of purified BmFHD. Buffers used are 50 mM HEPES-NaOH (6.5–8.0), 50 mM Tris-HCl (7.5–9.0) and 50 mM sodium borate (8.5–10.0). (C) Activity-substrate concentration plot of the purified BmFHD with 13*S*-HPOT. The activity with 13*S*-HPOT was evaluated by initial rate (for 5 sec from the onset of reaction) in 50 mM sodium borate buffer (pH 9.0). *inset*: The double-reciprocal plot of the data. (D) Purified BmFHD (60 ng) was incubated with 20 μM 13-HPOT in 50 mM sodium borate buffer (pH 9.0). At 50 and 90 sec after the start of reaction, 60 ng of purified BmFHD was repeatedly added (indicated with arrows). Inset: A semi-logarithmic plot of the remaining activity at the time indicated. **(**E**)** Purified BmFHD (60 ng) was incubated with 20 μM 13*S*-HPOT in 50 mM sodium borate buffer (pH 9.0). At 200 sec after the start of reaction, 20 μM 13*S*-HPOT was added again (indicated with arrows).

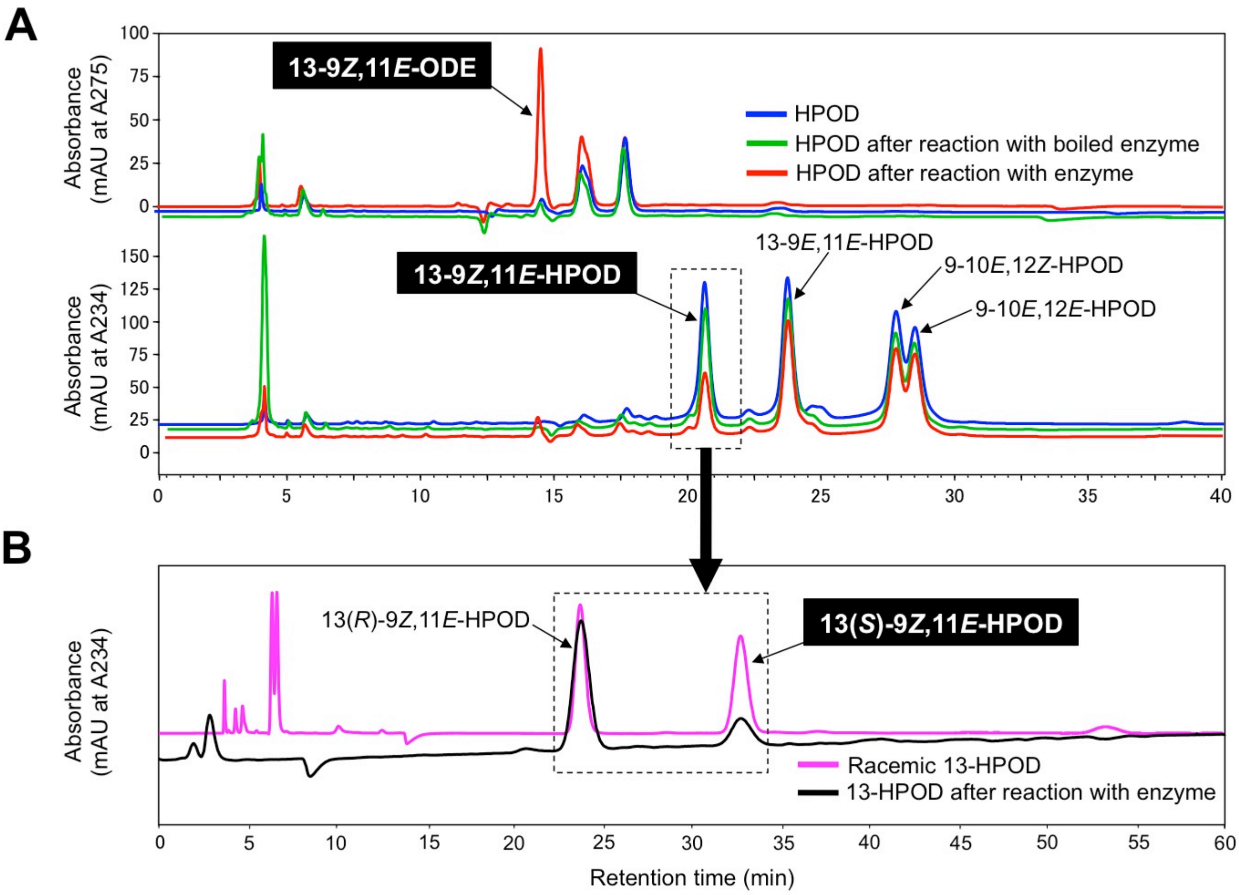

Supplementary Fig. 7. HPLC analysis of the substrates remaining after the reaction of purified BmFHD with HPOD mixture prepared via autooxidation. **(**A**)** The chromatograms on straight phase-HPLC of HPOD before reaction (blue), after reaction with heat-denatured BmFHD (green) and after reaction with active BmFHD (red) are shown. The chromatograms that were drawn by following A234 are shown at the bottom, and those drawn using A275 are shown at the top. (B) The chiral phase-HPLC of 13(*R*/*S*)-hydroperoxy-(9*Z*,10*E*)-octadecadienoic acid (13-HPOD) before reaction with BmFHD (magenta) and after reaction with BmFHD (black). 13*R*/*S*-HPOD was fractionated with straight phase HPLC and delivered to chiral phase HPLC. Under HPLC conditions, the 13(*R*)-enantiomer eluted faster than 13(*S*)-enantiomer.

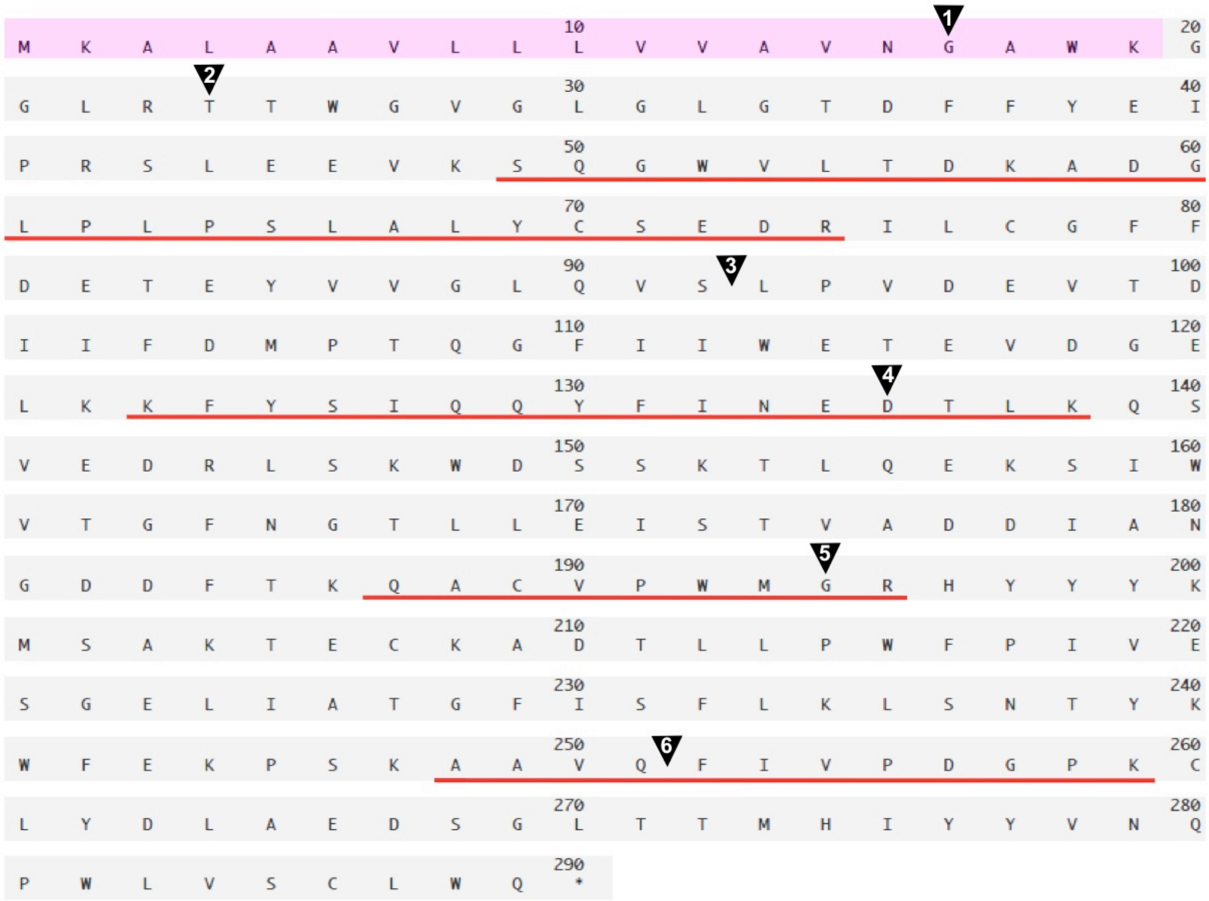

Supplementary Fig. 8. Amino acid sequence of BmFHD. The sequences identified with Mascot analyses are shown and underlined in red. The signal peptide can be identified as having a pink background. The exon/intron junctions predicted by SilkBase (http://silkbase.ab.a.u-tokyo.ac.jp) are shown with triangles.

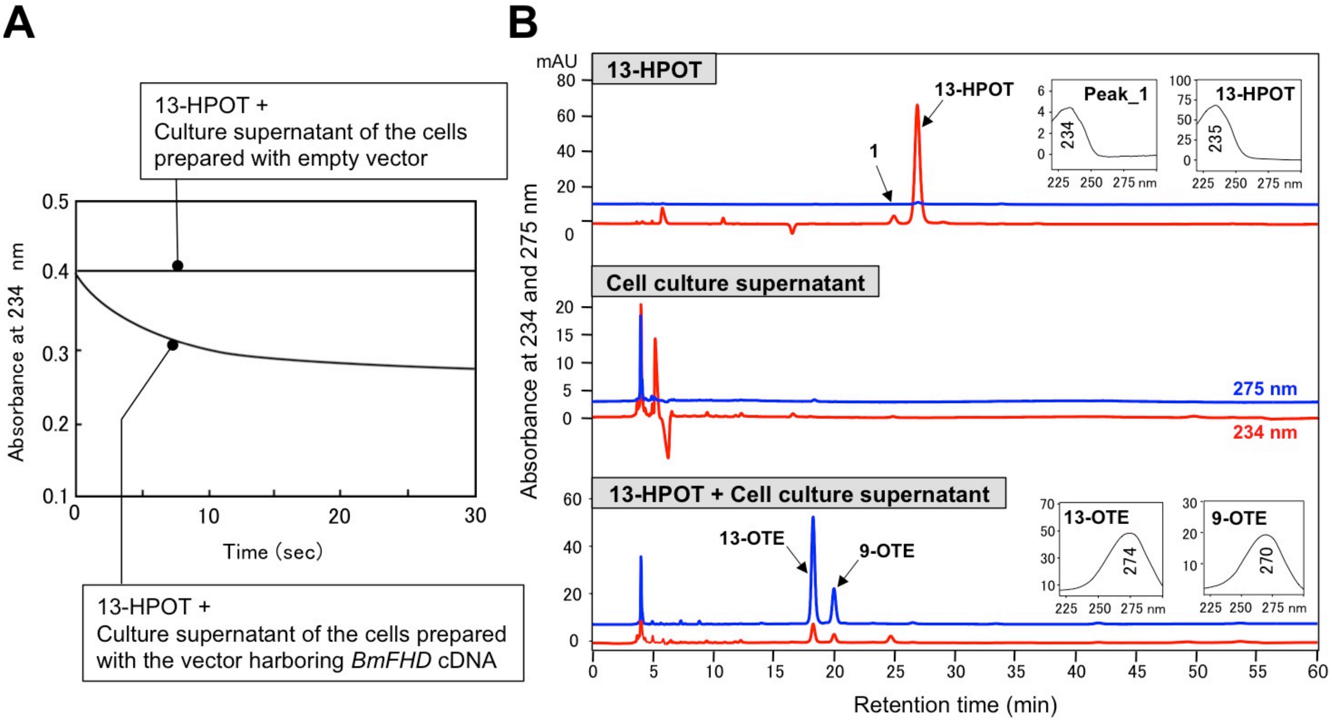

Supplementary Fig. 9. Reaction of the culture supernatant of BmN4 cells infected with the recombinant baculoviruses harboring BmFHD cDNA with 13*S*-HPOT. (A) Time course of decrease of A234 derived from 13*S*-HPOT (20 μM) by the cleared culture broth. (BS) The normal phase HPLC analysis of the products formed by the cell culture supernatant with 13*S*-HPOT. Products were monitored at A275 (blue line), and the remained substrates were monitored at A234 (red line). UV spectra of each peak are shown in the inset.

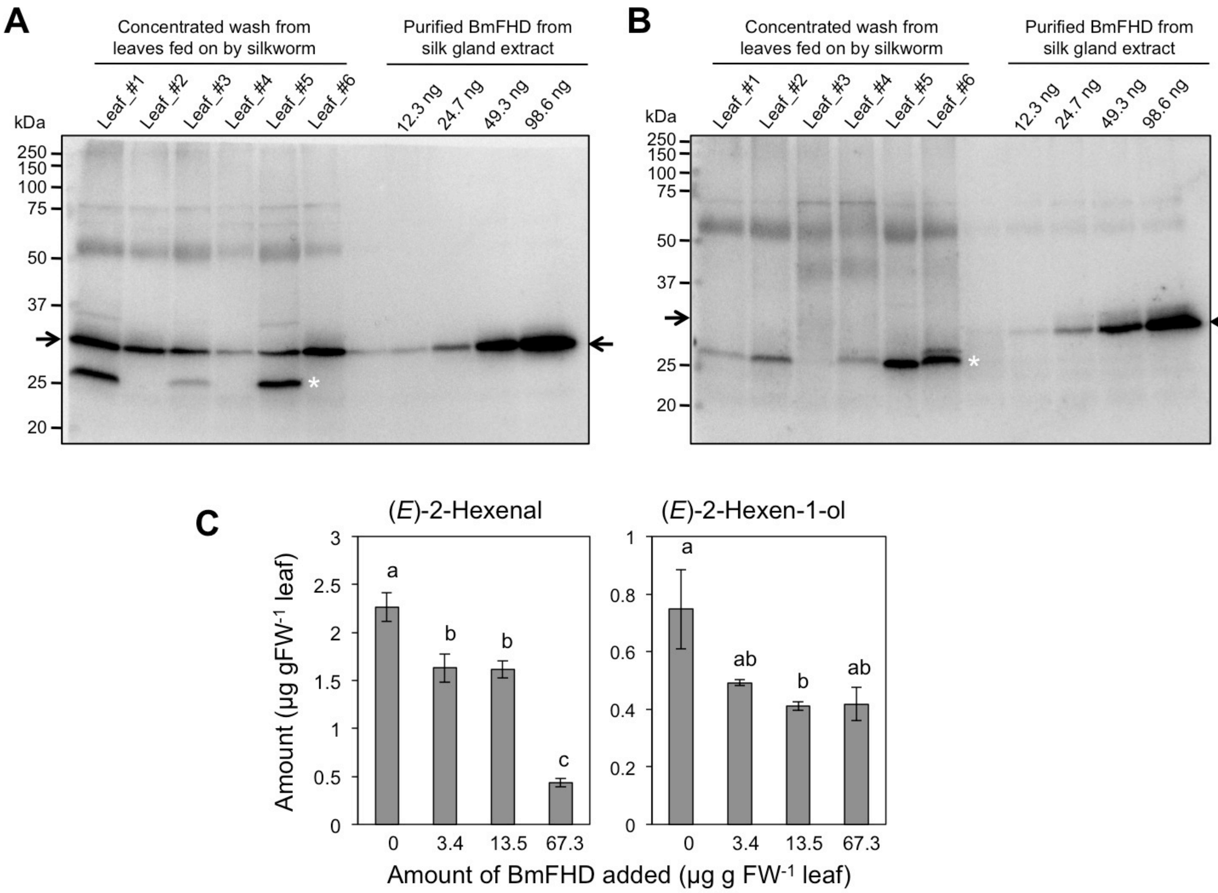

Supplementary Fig. 10. Confirmation of BmFHD left on the edges of a leaf fed on by silkworms. Rinse from the fed edge on a leaf (#1 to #6) made by a silkworm with a spinneret (A) and without a spinneret (B) was served for immunoblot analysis with purified BmFHD of a known amount. The position of BmFHD is indicated with the arrow. The bands indicated with the white asterisk are unknown. (C) The mulberry leaf powder was homogenized in the presence of a given amount of BmFHD purified from the silk gland of silkworms and incubated for 20 min to facilitate enzyme reaction to form (*E*)-2-hexenal (left) and (*E*)-2-hexen-1-ol (right). Averages with error bars (SE, $n = 4$, technical replicate) are shown. Different letters above the bars indicate significant differences among treatments ($P < 0.05$, GLM followed by Holm's P-value adjustment).

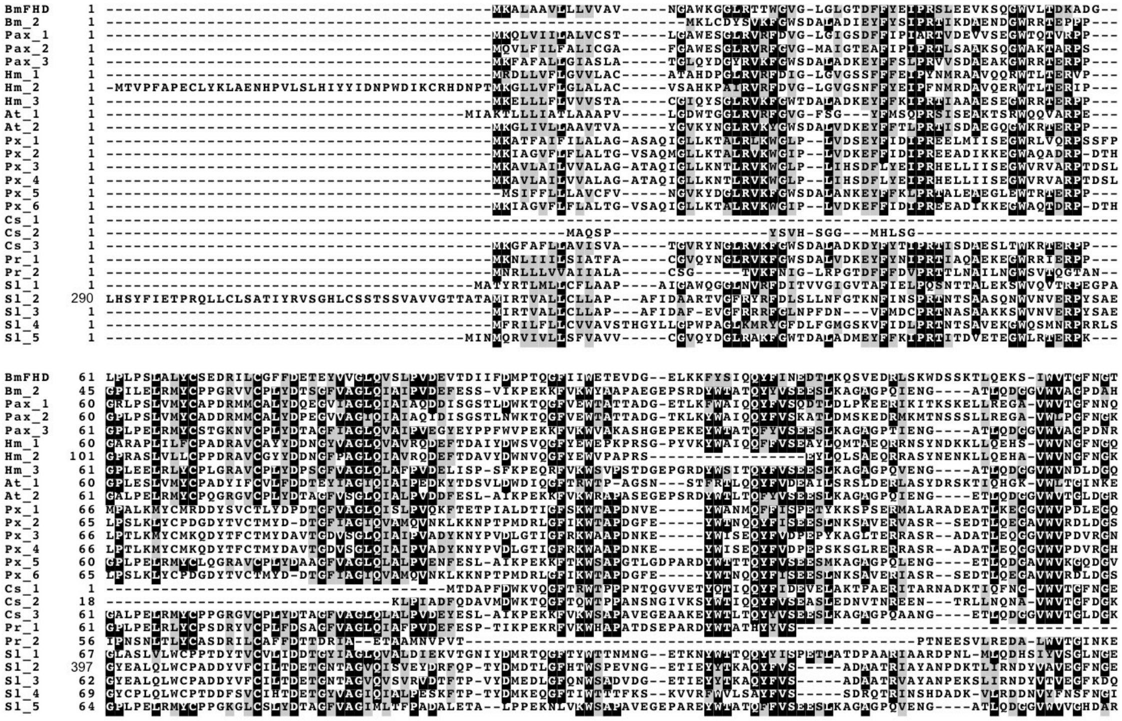

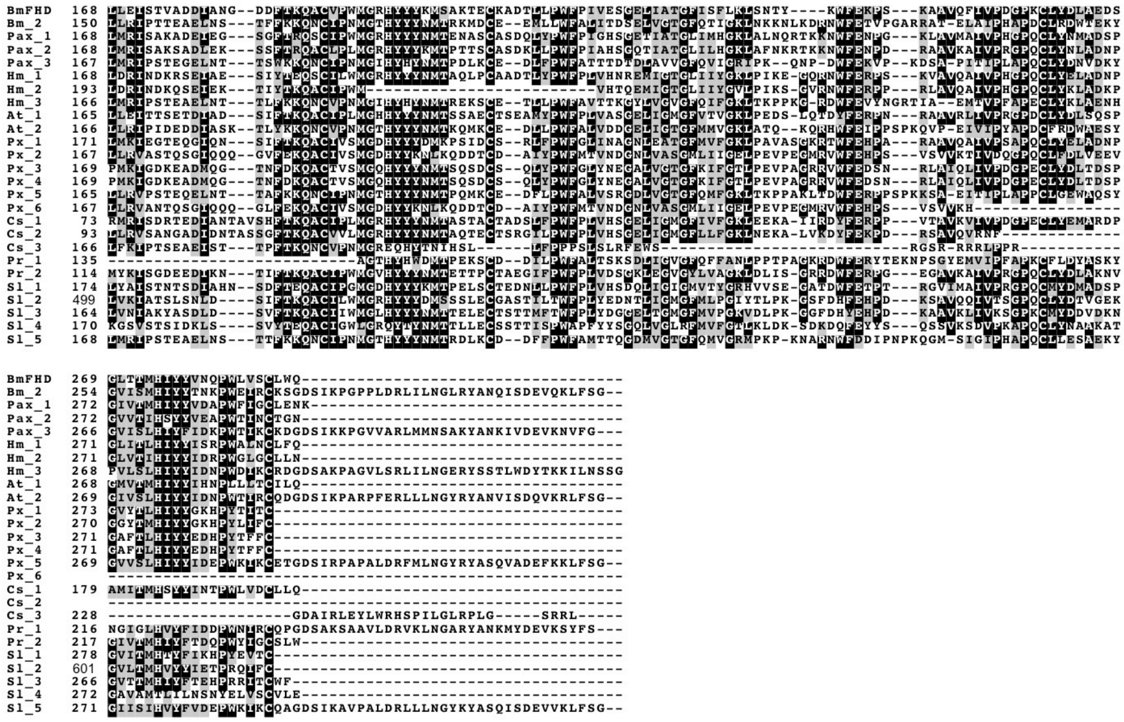

Supplementary Fig. 11. Multiple alignment of FHD like proteins in Lepidoptera insects used in phylogenetic tree analysis. Multiple alignment of amino acid sequences were performed using ClustalW alignment software in MEGA5. Gaps are indicated by dashes, letters with black background are identical amino acids, and letters with gray background are similar amino acids. Amino acid sequences are BmFHD: LC259005, Bm_2: XP004921895, Pax_1:

XP013171137, Pax_2: XP013170622, Pax_3: XP013171136, Hm_1: HMEL037892g1.t1, Hm_2: HMEL016940-PA, Hm_3: HMEL037893g1.t1, At_1: XP013182923, At_2: XP013182934, Px_1: XP011552765, Px_2: XP011567647, Px_3: g11888.t1, Px_4: XP011551596, Px_5: XP011552764, Px_6: XP011553122, Cs_1: CSUOGS104071-PA, Cs_2: CSUOGS108782-PA, Cs_3: CSUOGS107732-PA, Pr_1: genscan-scaff143-1, Pr_2: genscan-scaff143-2, Sl_1: XP022817470, Sl_2: XP022817942, Sl_3: XP022817893, Sl_4: XP022817879, Sl_5: XP022817709. Sl_2: XP022817942 has two ranges showing homology to BmFHD in one open reading frame, and the range corresponding to the C-terminal half was used for alignment.

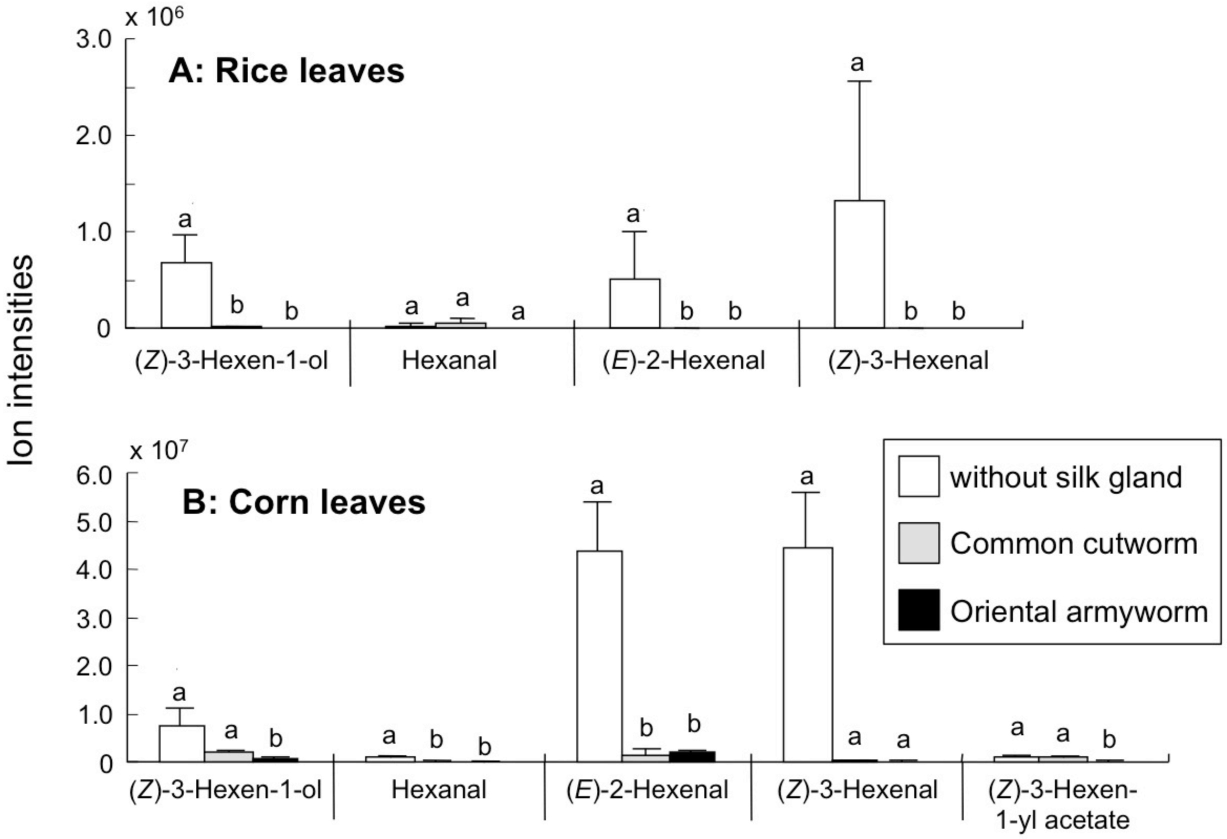

Supplementary Fig. 12. Effect of extract prepared from silk gland of oriental armyworms (*Mythimna separata*) and common cutworms (*Spodoptera litura*) on the production of green leaf volatiles in the homogenates of rice (*Oryza sativa*) or corn (*Zea mays*) leaves. Averages with error bars (SE, $n = 3$) are shown. Different letters above the bars indicate significant differences among samples ($P < 0.05$, GLM followed by Holm's P-value adjustment).

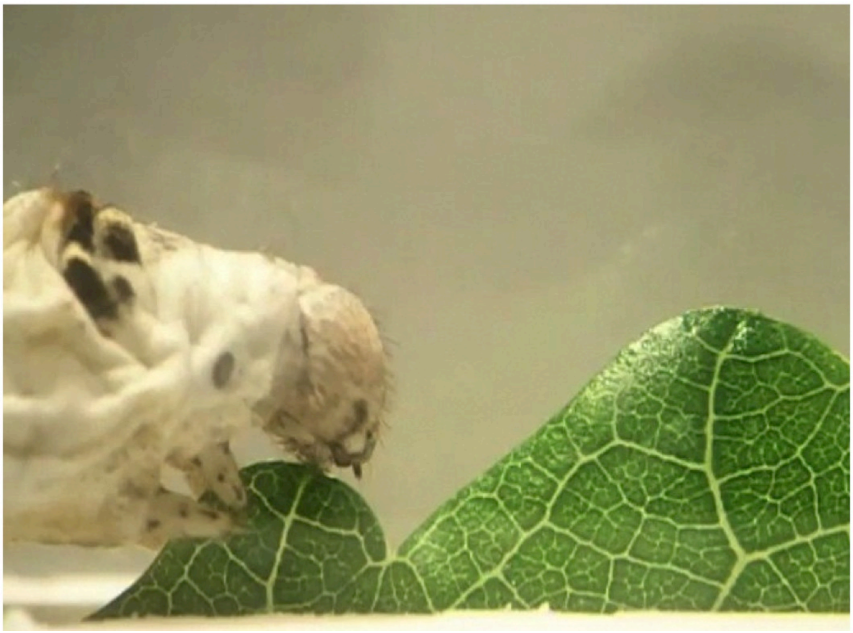

Supplementary Movie 1. A silkworm feeding on a mulberry leaf. A movie captured a silkworm, *Bombyx mori,* feeding on a mulberry leaf, *Morus alba*. Initial 13 sec is the real time movie, and after 13 sec a double-speed movie is shown.
